# CAM: an alignment-free method to recover phylogenies using codon aversion motifs

**DOI:** 10.7717/peerj.6984

**Published:** 2019-06-04

**Authors:** Justin B. Miller, Lauren M. McKinnon, Michael F. Whiting, Perry G. Ridge

**Affiliations:** 1 Department of Biology, Brigham Young University, Provo, UT, United States of America; 2 Brigham Young University, M.L. Bean Museum, Provo, UT, United States of America

**Keywords:** Phylogeny, Codon usage bias, Alignment-free, Codon aversion, Tree of life, Taxonomy, Maximum likelihood, Phylogenomics, Phylogenetics, Systematics

## Abstract

**Background:**

Common phylogenomic approaches for recovering phylogenies are often time-consuming and require annotations for orthologous gene relationships that are not always available. In contrast, alignment-free phylogenomic approaches typically use structure and oligomer frequencies to calculate pairwise distances between species. We have developed an approach to quickly calculate distances between species based on codon aversion.

**Methods:**

Utilizing a novel alignment-free character state, we present CAM, an alignment-free approach to recover phylogenies by comparing differences in codon aversion motifs (i.e., the set of unused codons within each gene) across all genes within a species. Synonymous codon usage is non-random and differs between organisms, between genes, and even within a single gene, and many genes do not use all possible codons. We report a comprehensive analysis of codon aversion within 229,742,339 genes from 23,428 species across all kingdoms of life, and we provide an alignment-free framework for its use in a phylogenetic construct. For each species, we first construct a set of codon aversion motifs spanning all genes within that species. We define the pairwise distance between two species, A and B, as one minus the number of shared codon aversion motifs divided by the total codon aversion motifs of the species, A or B, containing the fewest motifs. This approach allows us to calculate pairwise distances even when substantial differences in the number of genes or a high rate of divergence between species exists. Finally, we use neighbor-joining to recover phylogenies.

**Results:**

Using the Open Tree of Life and NCBI Taxonomy Database as expected phylogenies, our approach compares well, recovering phylogenies that largely match expected trees and are comparable to trees recovered using maximum likelihood and other alignment-free approaches. Our technique is much faster than maximum likelihood and similar in accuracy to other alignment-free approaches. Therefore, we propose that codon aversion be considered a phylogenetically conserved character that may be used in future phylogenomic studies.

**Availability:**

CAM, documentation, and test files are freely available on GitHub at https://github.com/ridgelab/cam.

## Introduction

Phylogenies allow biologists to analyze similar characters between species by providing an evolutionary framework to infer homology (Haszprunar, 1992; Soltis & Soltis, 2003). Although Next Generation Sequencing (NGS) facilitates placement of novel species on the Tree of Life, many regions of the genome display contradictory phylogenetic signals (Philippe et al., 2011). Furthermore, typical alignment-based phylogenetic methods require ortholog annotations to recover the phylogeny, and assembled genes without orthologous pairs provide no information for species relatedness using a traditional approach (Pais et al., 2014). Annotating a genome with orthologous relationships can often be costly and time-consuming, and some genes are currently impossible to annotate (Yandell & Ence, 2012). As complete genomes of more non-model organisms become available, correctly identifying orthologs will continue to impede accurate identification of taxonomic relationships. Common errors in recovering phylogenies include incorrect ortholog identification, erroneous alignments, and model violations for the phylogenetic tree reconstruction method (Philippe et al., 2011).

Alignment-free approaches were developed to address these, and other, issues. Since alignment-free methods do not use an alignment at any point in the algorithm, they can recover phylogenetic relationships even when recombination renders an alignment impossible (Zielezinski et al., 2017). Additionally, alignment-free algorithms are computationally less expensive, generally computed in linear time (Bonham-Carter, Steele & Bastola, 2014), are not subject to potential errors in orthology (Zielezinski et al., 2017), are resistant to shuffling and recombination events (Vinga, 2014), and are not dependent on assumptions regarding the correlation between sequence changes and evolutionary time (Zielezinski et al., 2017).

Alignment-free methods are based on sets of short oligonucleotides taken from the genome to infer phylogenies and often produce similar results as traditional methods (Chapus et al., 2005). The basic principle behind alignment-free phylogenetic tree reconstruction techniques is that genomic subsequences exhibit similar characteristics as the whole genome (Deschavanne et al., 1999). These genomic signatures are most prominent in highly divergent species arising from deep phylogenetic splits (Edwards et al., 2002). For example, since oligomer mutation rates vary dramatically between taxonomic groups, certain simple sequence repeats (SSRs) and long interspersed elements (LINEs) can sometimes be used to recover phylogenies (Shedlock et al., 2007).

More than 100 alignment-free methods have been developed. These methods use a widespread variety of approaches to make phylogenetic inferences. However, most methods are based on one of three principles: the frequencies of words of a certain length, the match lengths between sequences, or the calculation of informational content between two sequences (Zielezinski et al., 2017; Haubold, 2014). Additionally, novel approaches create “micro-alignments” to compare sequences. In our analysis, we limit our search space to coding sequences and compare the codon usages between species, ignoring all gene name annotations. We compare our algorithm to the word-based approaches, FFP (Jun etal., 2010; Sims et al., 2009) and CVTree (Zuo & Hao, 2015), the match-length approaches, ACS (Ulitsky et al., 2006), KMACS (Leimeister & Morgenstern, 2014), and Kr (Haubold et al., 2009), and the micro-alignment based approaches, Co-phylog (Yi & Jin, 2013), FSWM (Leimeister, Sohrabi-Jahromi & Morgenstern, 2017), and andi (Haubold, Klotzl & Pfaffelhuber, 2015). In addition to these comparisons with previous alignment-free techniques, we also provide a comparison with Maximum Likelihood, a common alignment-based technique. We analyze the performances of these algorithms based on accuracy and computational runtime.

Our approach exploits the Central Dogma of biology: three consecutive nucleotides of coding DNA, called codons, are used as a template for protein translation, where each codon encodes a single amino acid (Crick, 1970). The genetic code is degenerate because 64 canonical codons are used to form 20 amino acids and the stop signal (Crick et al., 1961). Gene expression is fine-tuned, in part, by the skewed occurrence of certain codons over others, called codon usage bias, because some codons are translated more efficiently than others (Quax et al., 2015). Differences in codon translational efficiencies are explained by unequal tRNA expression within different species and tissues, limiting the supply of anticodons directly complementing the codons (Quax et al., 2015). Complete codon aversion (i.e., when a codon is not used in a gene) can also be advantageous in certain genes, and is phylogenetically conserved within orthologs (Miller et al., 2017a). A significant portion of synonymous codon usage can also be explained by GC-biased gene conversion (gBGC), which occurs when transmission of GC alleles is favored over AT alleles during meiotic recombination (Duret & Galtier, 2009).

Our research explores the conservation of codon aversion and determines if sets of codon aversion motifs (i.e., the set of codons not used in each gene) are phylogenetically conserved. We also analyze amino acid aversion across all taxonomic groups, and we compare its phylogenetic conservation to that of codon aversion. We present a novel alignment-free algorithm, CAM, which we use to recover a phylogeny using the codon aversion or amino acid aversion of 229,742,339 genes from 23,428 species across the Open Tree of Life (OTL) (Hinchliff et al., 2015) and the NCBI taxonomy (Sayers et al., 2012; Sayers et al., 2011; Sayers et al., 2010; Sayers et al., 2009). CAM determines phylogenetic relationships by using only the overall differences in codon aversion within each gene across all available genes from a given species. Therefore, CAM does not require orthologous gene annotations. Our results suggest that codon and amino acid aversion patterns are conserved across all genes within a species and can be utilized to reconstruct phylogenetic trees without a sequence alignment.

## Materials & Methods

### Defining codon aversion motifs

We define a codon aversion motif as a set of codons that are not present in an individual gene. For example, a gene that uses all codons except for AAA and ATA would have a codon aversion motif of (AAA, ATA). We construct codon aversion motifs for each gene in a species, considering only each unique motif. For example, consider a species with four genes that have the following codon aversion motifs: (AAA, ATA), (AAA, ACG, CTC), (AAA, ATA), and (CGC). For this species, we would construct the following set of unique motifs: {(AAA, ATA), (AAA, ACG, CTC), (CGC)}. We constructed codon aversion motifs for all available genes of each species. Each gene was considered with equal weight, regardless of any orthologous annotations.

### Defining amino acid aversion motifs

Similar to codon aversion motifs, we also calculated amino acid aversion motifs. We first translated the DNA/RNA sequences to protein sequences. We then used the same process mentioned above to make sets of unused amino acids from each gene. After constructing amino acid/codon aversion motifs, all analyses are identical.

### Distance calculation and implementation

We constructed codon aversion motifs using all available genes in each species. Each gene, both annotated and unannotated, was given equal weight in our algorithm. We use differences in sets of codon aversion motifs found in each species to calculate the phylogenetic distances between species.

We calculate the pairwise distance between two species*, A* and *B*, as one minus the proportion of shared codon aversion motifs between the species. We define overlapping motifs as the intersection of codon aversion motifs in the two sets (i.e., codon aversion motifs that are found in both species). It is expected that a higher number of overlapping motifs will be present in closely related species because codon aversion is phylogenetically conserved in orthologs (Miller et al., 2017a). The proportion of shared codon aversion motifs is calculated by dividing the number of overlapping motifs between the two species by the number of possible overlapping motifs, where the number of possible overlapping motifs is defined as the number of motifs in the set, for species *A* or species *B*, containing the fewest motifs. We therefore calculate distances between two species, A and B, with sets of codon aversion motifs, *a* and *b*, respectively, with the following equation:

\begin{eqnarray*}Dist(A,B)=1- \frac{{|}a\cap b{|}}{\min \nolimits ({|}a{|},{|}b{|})} . \end{eqnarray*}

**Figure 1:**
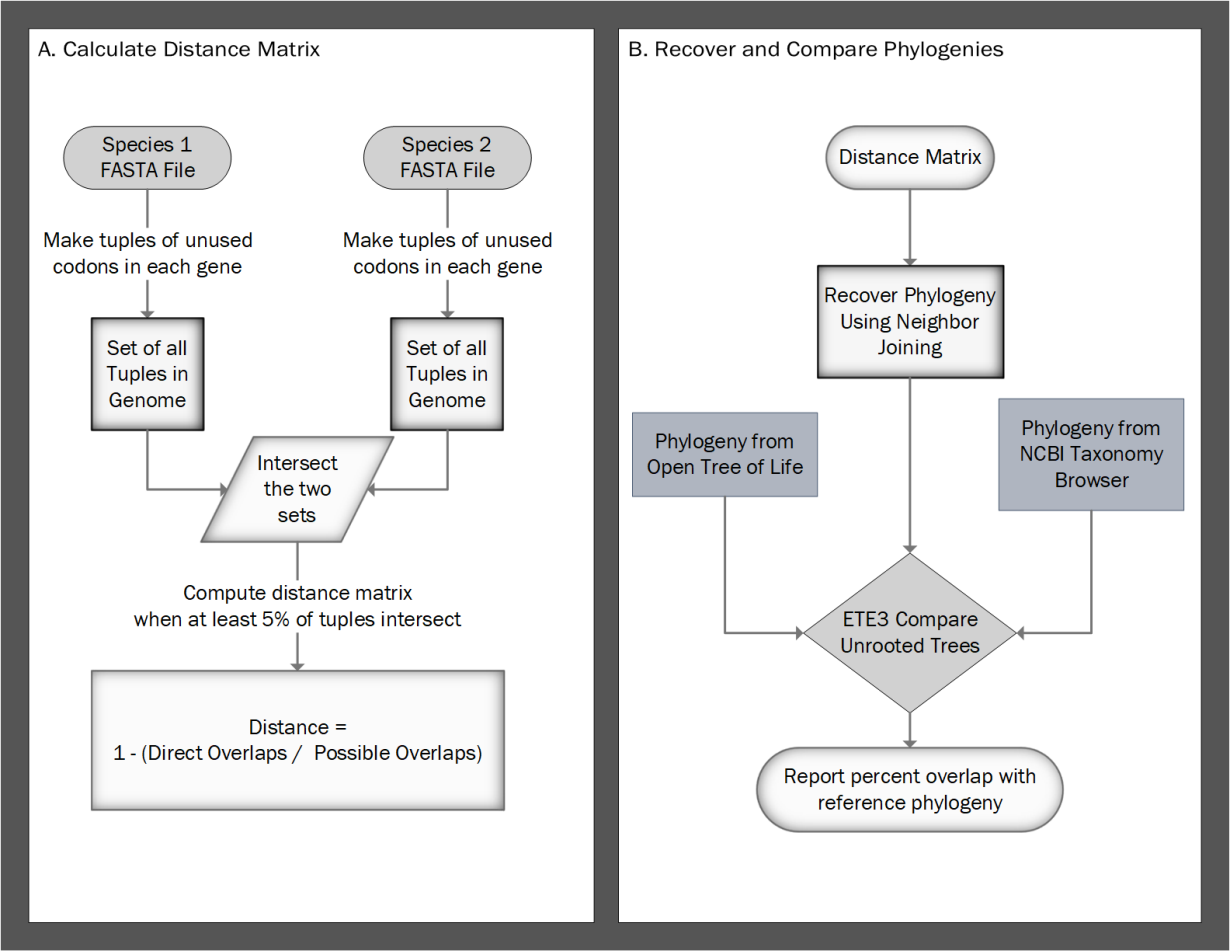
Flow charts for calculating the distance matrix and comparing the recovered phylogenies. (A) Calculate Distance Matrix: Start with two FASTA files of the DNA coding sequences of two species. For each species, find the unused codons within each gene, alphabetize them, and make those codons into a tuple. Add the tuple to an unordered set for that species. The distance is calculated by dividing the number of tuples in the intersection of the two sets by the minimum number of tuples in the two original sets. (B) Recover and Compare Phylogenies: From the distance matrix, use neighbor-joining to recover a phylogeny. We do not use a model of evolution to compute distances because distance is a function of the number of shared codon aversion motifs within a species. This technique allows a fair comparison of diverse or unknown species. Using the compare method within the Environment for Tree Exploration (ETE3), we then compare the unrooted tree with the OTL and the NCBI taxonomy. Finally, we report the percentage of the phylogenies that overlap.

This approach allows us to calculate pairwise distances (with a maximum distance of one), where smaller distances reflect species that share a large proportion of codon aversion motifs, and larger distances reflect species that share few codon aversion motifs. We also require that 5% of motifs between species overlap to limit any bias due to a small genome (e.g., it would not be unusual if a species with five genes has at least one codon usage motif that randomly overlaps with a motif from a species with 20,000 genes without directly inheriting 20% of its motifs from the same most recent common ancestor). This process is depicted in Fig. 1. We developed CAM in Python 3.5. CAM takes as input any number of species FASTA files, and creates a matrix of distances between species based on either codon aversion or amino acid aversion.

The most common way to run CAM is by using the following command, where ${DIR} is a directory with all compressed or uncompressed species FASTA files, one for each species, and ${MATRIX} is the path to a distance matrix that will be created:

python cam.py -i ${DIR}/* >${MATRIX}

For a summary of optional parameters when running CAM, see Supplemental Information 1.

### Phylogeny reconstruction

After the distance matrix was created, we use a Biopython (Talevich et al., 2012) implementation of neighbor-joining to recover the phylogenetic tree. Neighbor-joining was used to combine the pairwise species distances because each pairwise distance represented a distance based on codon aversion motifs present in a species, not homologous locations of the codon aversion motifs. We provide a Python script, makeNewick.py, that calculates the phylogenetic tree from the output matrix created by CAM using the following command:

python makeNewick.py -i ${MATRIX} -o ${OUTPUT}

All algorithms, with accompanying README and test files, are freely available from GitHub at: https://github.com/ridgelab/cam.

### Data collection and processing

We downloaded all coding sequences (CDS) from the National Center for Biotechnology Information (NCBI) in September, 2017 (Pruitt et al., 2014; Pruitt et al., 2000; Wheeler et al., 2007). The CDS regions of the reference genomes were derived from the most common alleles within each species (Pruitt et al., 2000; Wheeler et al., 2007). When multiple transcript isoforms were annotated, we used the longest isoform in order to include the most possible codons used in a gene. Additionally, we removed any annotated exceptions from the gene dataset (e.g., translational exceptions, unclassified transcription discrepancies, suspected errors, etc.). Most sequences do not have annotated exceptions, and these filters removed fewer than 5% of sequences from each species. Partial gene annotations were included in the analysis. Although not present in most species, some species included large numbers of partial gene sequences, so we included partial gene sequences in the main analysis (See Fig. S1 for the percentage of partial protein sequences in each taxonomic group). We also compared the phylogenies recovered with and without partial gene sequences to determine the robustness of this method to partial gene inclusion.

### Data analyzed

Our analyses included 23,428 species, which were divided into the following taxonomic groups based on annotations within the NCBI database: 418 archaea, 15,058 bacteria, 234 fungi, 149 invertebrates, 89 plants, 75 protozoa, 107 mammalian vertebrates, 123 other vertebrates, and 7,233 viruses. Sixty-eight species are included in both bacteria and viruses because they are annotated in both taxonomic groups in RefSeq. Using CAM, we reconstructed phylogenetic trees for each of these taxonomic groups. We also reconstructed a phylogenetic tree for all 23,428 species.

### Reference phylogenies

In order to determine the accuracy of our phylogenetic trees, we compared them to reference trees from both the OTL and the NCBI Taxonomy Browser. Although the NCBI Taxonomy Browser is not considered a primary source for taxonomic phylogenetic information because it gathers phylogenetic annotations from many sources, it provides useful information for our analyses because it includes more species than the OTL. Although the OTL and the NCBI reference trees are biased by the tree reconstruction methods originally used to assemble the trees, they provide comprehensive trees spanning all species that can be used in our comparisons. Both trees combine the results from multiple studies and are based on multiple phylogenomic approaches. We assessed the accuracy of codon aversion by comparing recovered phylogenies to trees from each of these databases.

### Extracting phylogenies from the Open Tree of Life

We used the OTL documentation for programmatically inferring subtrees to develop a Python 3.5 program, getOTLtree.py, that retrieves subtrees from the OTL. Although other OTL parsers, such as ROTL (Michonneau, Brown & Winter, 2015), are available, getOTLtree allows users to obtain a subtree of any number of species from the OTL in a single step. Inferring subtrees from a set of species requires accessing the OTL database twice: first to retrieve OTL Taxonomy Identifiers (OTT ids) for each species, and second to retrieve the phylogenetic tree. getOTLtree does both commands in a single step at runtime, prompting the user to manually select the correct domain of life when duplicates are found in the OTL database (e.g., *Nannospalax galili* is listed as a eukaryote [OTT id: 207281] and as a bacterium [OTT id: 5909124]). Furthermore, we account for the OTL command, match_names, which limits identical matching of species to 1,000 names, by combining results from multiple queries of fewer than 1,000 species. This process makes large-scale species analyses easier and takes only a few seconds to extract a phylogeny of 2,000 species on a single processing core. If each species is listed on a different line (or CSV or Newick format) in a file called ${INPUT}, the typical usage for extracting the tree from the OTL is:

python getOTLtree.py -i ${INPUT}

**Figure 2:**
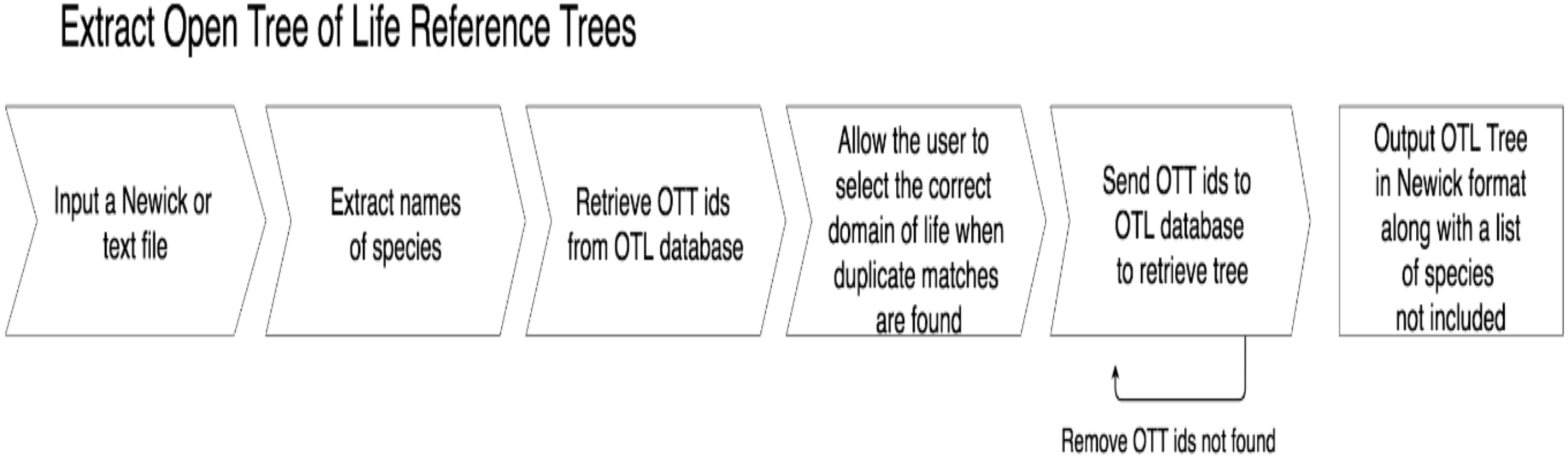
A flow chart depicting the process getOTLtree takes to infer a subtree phylogeny from the OTL. All steps are done with a single command at runtime.

getOTLtree, accompanying test files, and a README with more detailed explanations of how to run the program with different parameters are also available in the GitHub repository at https://github.com/ridgelab/cam. A summary of the process used by getOTLtree is depicted in Fig. 2.

### Extracting phylogenies from the NCBI Taxonomy Browser

The NCBI Taxonomy Browser (https://www.ncbi.nlm.nih.gov/Taxonomy/CommonTree/wwwcmt.cgi) has many tools to enable large queries of its database. We opted to include unranked taxa in our analyses to maximize the number of included species. We downloaded the phylogeny in PHYLIP (Felsenstein, 1989) format directly from the website, and used the extracted phylogenies in our analyses.

### Tree comparison

We used the ete-compare module from the Environment for Tree Exploration toolkit (ETE3) (Huerta-Cepas, Dopazo & Gabaldon, 2010; Huerta-Cepas, Serra & Bork, 2016) to quantify the similarity between the tree constructed using codon aversion and the corresponding reference trees from the OTL and the NCBI taxonomy. The following command calculates edge similarity of an unrooted tree, where ${INPUT} is the path to the recovered tree and ${REF} is the path to the reference tree from the OTL or the NCBI taxonomy:

ete3 compare -t {INPUT} -r {REF}–unrooted

We selected the percentage of edge similarity (i.e., the number of branches in one tree that are present in the other tree) to compute the topological distance between the trees. This metric was selected based on the following criteria: capability to efficiently compare very large trees, capability to compare unrooted trees (neighbor-joining is unrooted by definition (Saitou & Nei, 1987) and we wanted to account for potential variations at the root node in the reference tree), and capability to compare trees with polytomies. Although several tree-comparison metrics exist, many suffer from problems ranging from high computational cost to lack of robustness (Lin, Rajan & Moret, 2012). Advantages for using the percentage of edge similarity metric from the compare method in ETE3 include: clarity in comparing the output as a percentage of congruent branches between trees, optimization for large datasets, capability to compare unrooted trees, and robustness to polytomies (Huerta-Cepas, Serra & Bork, 2016). The advantages and disadvantages of several common tree comparison techniques are listed in Table S1.

### Validation using maximum likelihood

Since maximum likelihood (Felsenstein, 1981) has been widely used to construct the current version of the OTL, there is a potential confirmation bias when comparing it to the OTL (i.e., it is likely to have an artificially high percent overlap with the species relationships found in the OTL since it was used to create the OTL). However, it is still widely used and should be evaluated against our alignment-free technique. Using ortholog annotations from NCBI, which combines annotations from species-specific nomenclature committees (e.g., the HUGO Gene Nomenclature Committee (HGNC) (Gray et al., 2015)), NCBI staff curations, and the NCBI annotation pipeline, we extracted the most commonly used orthologs in each taxonomic group. Although we performed no formal tests for orthology, in cases where duplicated genes with the same gene names existed (e.g., RPS4 in the mitochondrion and rps4 in the chloroplast are both listed in *Arabidopsis thaliana*), both genes were removed. After this filtering, we performed a multiple sequence alignment (MSA) on the DNA sequences of each ortholog using the following CLUSTAL OMEGA (Sievers & Higgins, 2018) command:

clustalo -i ${INPUT}>${OUTPUT}

We used CLUSTAL OMEGA because it performed very well in full-length sequence comparisons Pais et al. (2014), and we used full-length gene sequences in our analyses. After each MSA was completed, we created a super-matrix by concatenating the alignments from all orthologs for each species (if an ortholog was not annotated for a species, all nucleotide characters for that ortholog were expressed as “-” for that species). After the super-matrix was created, we used the following IQ-TREE (Nguyen et al., 2015) command to automatically choose the correct model (Posada & Crandall, 1998) and perform maximum likelihood to recover the phylogeny:

iqtree -s ${INPUT} -m TEST -pre ${OUTPUT}

The recovered phylogeny was then compared to the OTL and the NCBI Taxonomy using the unrooted compare method from ETE3 to identify branch similarities.

### Comparison with traditional k-mer approach

One alignment-free technique to recover phylogenies is to create a feature frequency profile (FFP) which consists of counting the occurrences of different k-mers and comparing those profiles between species (Jun etal., 2010; Sims et al., 2009). Although FFP is often used on the whole genome, it can also be used on the proteome (Jun etal., 2010), which allowed us to do a direct comparison of this approach using our dataset, which consists of all CDS regions. All analyses were done using the step-by-step procedures outlined in the FFP software README. Since the FFP software requires uncompressed data, we uncompressed all FASTA files before conducting the analysis. Preprocessing time was not included in the comparison results.

We included all species FASTA files in a single directory, ${DIR}. If all species names are shorter than 10 characters, they can be included in a single file called ${SPECIES}. However, if any species names are longer than 10 characters, then a list of numbers (IDs) can be substituted for the species names. We used unique IDs for this step and then converted them back to species names after the tree was recovered. We used the recommended command from the FFP README (https://sourceforge.net/projects/ffp-phylogeny/files/Documentation/) to create the distance matrix, ${MATRIX}:

ffpry -l 5 ${DIR}/* — ffpcol — ffprwn — ffpjsd -p ${SPECIES >${MATRIX}

### Comparison with CVTree approach

CVtree is an example of a word-based approach (Zuo & Hao, 2015). The algorithm uses composition vectors to compute frequencies of words of a given length. It then normalizes these frequencies by the expected frequencies predicted by random chance. Finally, it compares these frequencies between species to compute a distance.

We ran CVTree across each taxonomic group by following the procedure outlined in the CVTree README (https://github.com/ghzuo/CVTree). We first created a file containing the names of each species to be compared called ${SPECIESLIST}. We also created a directory of the species FASTA files called ${DIR}. We retained the default settings for word length, which counts words of lengths five, six, and seven. We then used the recommended command to compute the distance matrix, ${MATRIX}:

./build/bin/cvtree -g ffn -G ${DIR} -i ${SPECIESLIST} -t ${MATRIX}

### Comparison with Average Common Substring Approach (ACS)

ACS is based on substring match lengths (Ulitsky et al., 2006). This algorithm finds the longest substring, beginning at each index of a sequence, that is also found in a second sequence. They use the average of these matching substrings to calculate a distance.

We ran ACS using an implementation described by Leimeister & Morgenstern (2014), and can be found at http://kmacs.gobics.de/. This algorithm takes a single sequence as input for each species. In order to do a whole-genome analysis of the species, we first created an input FASTA file called ${INPUT} for each dataset containing a single sequence for each species. We created this single sequence by concatenating all genes together, separating each gene by ten ‘N’ characters to limit potential biases based on the order that the genes were concatenated. We then followed the steps found in the ACS README file. This implementation allows the user to specify a k-value for the number of mismatches allowed, we ran the algorithm with a k-value of 0, which calculates ACS distances. We used the recommended command to compute the distance matrix:

./kmacs ${INPUT} 0

### Comparison with K-mismatch Average Common Substring Approach (KMACS)

KMACS is another approach based on match lengths (Leimeister & Morgenstern, 2014). This algorithm is similar to ACS, but it differs by allowing k number of mismatches in the common substrings.

We ran KMACS using the same implementation that we used to compute ACS (http://kmacs.gobics.de/). We used the same input FASTA files, ${INPUT}, described in our ACS comparisons. Each input file contained a single sequence for each species. We ran KMACS with a k-value of 1, using the following command:

./kmacs ${INPUT} 1

### Comparison with Kr approach

Kr is also based on match lengths (Haubold et al., 2009). This algorithm estimates the number of mutations per site. It reduces the computational runtime of the algorithm by creating a generalized suffix tree of all input sequences to identify the match lengths.

We ran Kr using the steps outlined in the README (http://guanine.evolbio.mpg.de/kr/). We used the same input FASTA files for single sequences that were previously used in the ACS and KMACS comparisons (${INPUT}). We used the following command for each comparison:

./kr ${INPUT}

### Comparison with co-phylog

Co-Phylog is considered a novel alignment-free approach (Yi & Jin, 2013). Co-phylog creates “micro-alignments” that enclose a maximum of one mismatch across all species. Instead of conducting a global sequence alignment, co-phylog combines the mismatches from multiple local alignments into a single matrix that is then used to estimate a mutation rate.

We ran Co-Phylog using the steps found in the README (https://github.com/yhg926/co-phylog). The first step was to make “co-files” for each of the species FASTA files. We created co-files with the following command:

./fasta2co ${SPECIES_FASTA}${SPECIES_CO_FILE}

The second step was to use the directory of co-files, ${DIR}, to create a distance matrix called ${MATRIX}. We used the following command:

./co2dist ${DIR}>${MATRIX}

### Comparison with andi

Andi is another novel alignment-free approach (Haubold, Klotzl & Pfaffelhuber, 2015). Andi uses a similar approach to Co-phylog, but it allows the local “micro-alignments” to include more than a single mismatch. It searches for mismatches that are bracketed by long exact matches, referred to as *anchors*.

We ran andi using the steps found in the README (https://github.com/evolbioinf/andi/). We used as input each of the species FASTA files in our original dataset (${INPUT}). We ran andi using the default parameters. We also include the –join parameter to indicate that each sequence in the individual FASTA files is part of the same species. We performed this analysis with the following command:

./andi –join ${INPUT}

### Comparison with filtered spaced-word matches

Filtered spaced-word matches (FSWM) is another novel alignment-free approach that, similar to Co-Phylog and andi, finds matching-spaced words between sequences (Leimeister, Sohrabi-Jahromi & Morgenstern, 2017). It differs from these previous methods by accounting for pattern matches caused by random chance.

We ran FSWM using the steps found in the README (https://github.com/evolbioinf/andi/). We used the same input FASTA files, ${INPUT}, described in the ACS and KMACS comparisons because the input files are required to contain a single sequence for each species. We used the following recommended command to compute the distance matrix:

./fswm ${INPUT}

### Using neighbor-joining to infer phylogenetic trees

The methods above (FFP, CVTree, ACS, KMACS, Kr, Co-Phylog, andi, and FSWM) each created a distance matrix, ${MATRIX}, in PHYLIP format. We used the same Biopython implementation of the neighbor-joining algorithm that CAM used by specifying the PHYLIP input format option (-p) of makeNewick.py (provided in the GitHub repository for CAM):

python makeNewick.py -p -i ${MATRIX} -o ${OUTPUT}

After the Newick tree was recovered and the species IDs were converted back to species names, we compared the recovered tree with the OTL and the NCBI taxonomy using the unrooted compare method in ETE3.

## Results

### Frequency of codon aversion motifs

Since 64 codons exist, and each species typically uses only one of three possible stop codons and the one start codon per gene, there are 61 degrees of freedom (64 –2 unused stop codons –1 start codon), allowing for 2^61^ possible motifs. Similarly, amino acid aversion motifs have 20 degrees of freedom (for 20 amino acids), allowing for 2^20^ possible motifs. We observed 54,336,494 (∼2^26^) codon motifs across all genomes, with significant overlap between species (see Table 1). When including counts for multiple occurrences of a motif within the same species, there are still more than 5x as many completely unique motifs (i.e., motifs that occur in a single gene within a single species) as overlapping motifs (i.e., motifs that occur in multiple genes or multiple species) (See Figs. S2–S11). We also note that not all codons have equal probabilities of being present in a gene, and we show the frequency of codon aversion per codon within each taxonomic group in Figs. S12–S21. Although most genes use most codons, some genes exclude significantly more codons than others. Across all species, the mean number of codons not used within a sequence is 14.4819, with a standard deviation of 8.6881 codons. The number of codons included in each codon aversion motif is depicted in Figs. S22–S31. In Figs. S32–S41, we also show that relatively few motifs are present in more than a few genes.

**Table 1:** Unique tuples in each taxonomic group. Unique tuples were calculated by adding all tuples of unused codons from all genes within each species from a taxonomic group to a set, and then counting the number of elements in that set. The All group includes all species in the same analysis. Total (without all) sums the number of motifs and genes from each taxonomic group, calculated individually. Since most species in this analysis are bacteria, Total (without all and without bacteria) summed the values from each taxonomic group without including bacteria or all species combined. Note: 23,983 viral and bacterial genes overlap and 1,048,861 motifs span different taxonomic groups (difference between values in All and Total (without all).

Taxonomic group	Number of unique motifs	Number of genes	Average number of genes with a given motif
All	54,336,494	229,742,339	4.228
Archaea	1,057 898	1,903,114	1.799
Bacteria	49,177,047	215,581,296	4.384
Fungi	904,513	2,194,206	2.426
Invertebrates	951,901	2,153,164	2.262
Plants	1,009,268	2,510,219	2.487
Protozoa	510,582	841,682	1.648
Mammals	732,868	2,004,675	2.735
Other vertebrates	806,510	2,274,837	2.821
Viruses	234,768	303,129	1.291
Total (without all)	55,385,355	229,766,322	4.149
Total (without all and without bacteria)	5,159,447	14,161,043	2.745

### Trees constructed by CAM, amino acid motifs, maximum-Likelihood and alignment free techniques

We ran each alignment-free algorithm on a 24-core Intel Broadwell (2.4 GHz) compute node. For each analysis, we allowed the algorithms to run for a maximum of 3 days on 24 processing cores with a maximum of 256 GB of RAM. With these constraints, CAM, amino acid motifs, and FFP each recovered a tree for all 23,428 species. ACS, CVTree, andi, and FSWM recovered trees for most of the analyses. ACS and andi exceeded the time limitation for all species and bacteria. CVTree had a segmentation fault on comparisons for all species and bacteria. FSWM exceeded the memory limitation for all species and bacteria. KMACS exceeded the three-day time limit for all of the analyses except for protozoa. In addition, Co-phylog was not able to complete any of the analyses in the allotted time. Kr exceeded the maximum memory allocation for each analysis. Maximum likelihood recovered trees for most of the analyses, although insufficient ortholog annotations were available in bacterial species and all species. The maximum likelihood trees included relatively few fungi (25%), protozoa (32%), invertebrates (38%), and plants (67%) because many of the species did not have ortholog annotations. The NCBI taxonomy included almost all species found in RefSeq, missing only two archaea, 456 bacteria, and 188 viruses. Since the OTL does not include viruses, it contains significantly fewer species, with the inferred phylogeny containing only 12,337 species out of the possible 23,428 species. We show the number of species included in the phylogenies recovered by each algorithm in Table 2, excluding KMACS, Co-Phylog, and Kr which were unable to complete the analyses.

**Table 2:** Number of species included in phylogenies. For each algorithm, we report the number of species used to recover the phylogeny.

Taxonomic group	CAM	Amino acid motifs	FFP	CVTree	ACS	Andi	FSWM	Maximum likelihood	NCBI taxonomy	OTL
All	23,428	23,428	23,428	N/A	N/A	N/A	N/A	N/A	22,794	12,337
Archaea	418	418	418	418	418	418	418	418	416	362
Bacteria^a^	15,068	15,068	15,068	N/A	N/A	N/A	N/A	N/A	14,612	11,227
Fungi	234	234	234	232	232	232	232	58	234	214
Invertebrates	149	149	149	149	149	149	149	57	149	147
Plants	89	89	89	89	89	89	89	60	89	87
Protozoa	75	75	75	75	75	71	75	24	75	75
Mammals	107	107	107	107	107	107	107	100	107	105
Other vertebrates	123	123	123	123	123	123	123	118	123	120
Viruses^a^	7,233	7,233	7,233	6,996	7,230	6,996	6,996	N/A	7,045	N/A

**Notes.**

a Some species are included in both bacteria and viruses.

### Percent similarity compared to reference trees

We compared the recovered phylogenies from each of the algorithms with the reference phylogenies from the OTL (Table 3) and the NCBI taxonomy (Table 4). Of the CAM analyses, bacteria and viruses have the highest similarity with the reference phylogenies (84–91%), and invertebrates have the lowest similarity (60–70%). In most instances, amino acid aversion motifs performed comparably to codon aversion motifs when compared against the OTL and the NCBI taxonomy. However, the percent overlap between the NCBI taxonomy and amino acid aversion motifs in mammals, other vertebrates, and viruses was much lower than the percent overlap with CAM (9–25% lower). The same trend exists when comparing the recovered trees with the OTL, with amino acid motifs recovering 10–14% fewer species relationships than CAM. The other taxonomic groups did not appear to vary significantly between the recovered trees using amino acids or codons, with the difference between the two methods being −3% to +3% for the NCBI taxonomy and −5% to +2% different for the OTL. CAM and the other alignment-free algorithms all had similar percent similarities to the reference trees. There was no single algorithm that consistently had the highest percent similarity compared to the references. Maximum likelihood also recovered trees with comparable branch percent similarities with the alignment-free methods.

**Table 3:** Comparison to the OTL. Percent edge overlap of an unrooted tree comparison of each algorithm versus the established phylogeny from the OTL for each taxonomic group. Maximum likelihood could not compute a tree for bacteria or all species because insufficient ortholog annotations were available for the majority of these species. ACS, andi, and FSWM could not complete bacteria and all species analyses due to time or memory constraints.

Taxonomic group	CAM	Amino acid motifs	FFP	CVTree	ACS	Andi	FSWM	Maximum likelihood	NCBI taxonomy
All	82	84	83	N/A	N/A	N/A	N/A	N/A	95
Archaea	75	77	74	80	80	68	82	89	94
Bacteria	84	84	85	N/A	N/A	N/A	N/A	N/A	95
Fungi	69	67	67	73	75	65	69	65	91
Invertebrates	60	57	55	65	68	63	78	73	98
Plants	64	63	54	72	79	70	85	73	98
Protozoa	65	65	64	72	68	60	75	64	93
Mammals	77	63	52	69	90	95	94	93	99
Other vertebrates	66	56	54	68	76	81	80	81	94

**Table 4:** Comparison to the NCBI taxonomy. Percent edge overlap of an unrooted tree comparison of each algorithm versus the established phylogeny from the NCBI taxonomy for each taxonomic group. Maximum likelihood could not compute a tree for bacteria, viruses, or all species because insufficient ortholog annotations were available for the majority of these species. ACS, andi, and FSWM could not complete bacteria and all species analyses due to time or memory constraints.

Taxonomic Group	CAM	Amino acid motifs	FFP	CVTree	ACS	Andi	FSWM	Maximum likelihood
All	89	90	90	N/A	N/A	N/A	N/A	N/A
Archaea	81	84	80	85	86	76	89	92
Bacteria	91	90	91	N/A	N/A	N/A	N/A	N/A
Fungi	73	69	69	75	77	67	72	70
Invertebrates	70	68	65	75	78	71	70	78
Plants	71	70	61	80	84	78	92	79
Protozoa	72	71	72	82	78	68	85	73
Mammals	87	73	63	80	95	98	98	98
Other vertebrates	79	70	67	83	90	93	93	95
Viruses	90	65	91	91	92	89	60	N/A

As expected, the NCBI taxonomy and the OTL are highly similar (Table 3), although 6–9% of species relationships disagree outside of invertebrates, plants, and mammals. Even though the NCBI and OTL reference trees are similar to each other, our analyses lend support to the NCBI taxonomy in every taxonomic group –70 out of the 71 completed analyses reported phylogenies being 2–15% more similar to the NCBI taxonomy than the OTL.

We also ran the entire CAM analysis excluding partial sequences. Excluding partial genes had a minimal effect on the overall percent overlap with the OTL (minus 2% to plus 5% similarity) and the NCBI taxonomy (minus 2% to plus 3% similarity).

### Comparing algorithm runtimes

Table 5 shows the CPU runtime of each algorithm in hours. The alignment-free techniques had significantly faster runtimes than the maximum likelihood approach. FFP and CVTree consistently had the fastest runtimes. CAM and amino acid motifs also ran quickly with runtimes ranging from less than 2 minutes for the smaller datasets, such as protozoa, to approximately 20 hours for all species. Runtime was always longer for amino acid motifs than CAM because the DNA sequences were translated into protein sequences before being evaluated for amino acid usage. Runtimes for adni ranged from 1 to 2 hours for the smaller taxonomic groups excluding bacteria. ACS ran slightly slower with a range of 4 to 42 hours. FSWM was the slowest alignment-free method with CPU runtimes ranging from 20 to 63 hours, excluding bacteria. Maximum likelihood required between 2.5 and 200 hours of CPU time to compute a tree for each taxonomic group.

**Table 5:** CPU runtime of each algorithm in hours. CVTree and FFP were the fastest algorithms. CAM and Amino acid motifs had comparable runtimes and were faster than ACS, andi, FSWM, and maximum likelihood.

Taxonomic group	CAM	Amino acid motifs	FFP	CVTree	ACS	Andi	FSWM	Maximum likelihood
All	17.2794	20.2692	3.9072	N/A	N/A	N/A	N/A	N/A
Archaea	0.0667	0.1436	0.0408	0.0236	28.87	8.05	28.83	161.5
Bacteria	14.6994	17.4458	3.7442	N/A	N/A	N/A	N/A	N/A
Fungi	0.0783	0.2167	0.0294	0.0028	42.12	8.75	56.92	199.75
Invertebrates	0.0763	0.2126	0.0447	0.0150	28.75	5.88	54.93	2.5
Plants	0.0781	0.2211	0.0383	0.0217	22.17	4.21	49.77	6.0
Protozoa	0.0287	0.0833	0.0183	0.0078	4.88	1.01	20.65	4.0
Mammals	0.0718	0.2101	0.0294	0.0122	22.32	4.32	63.25	2.5
Other vertebrates	0.0872	0.2356	0.0322	0.0206	27.03	5.63	61.35	6.75
Viruses	0.1028	0.1161	0.1019	0.2906	42.53	12.67	6.03	N/A

Although the maximum likelihood analysis was not possible on bacteria or all species because insufficient ortholog gene annotations exist to accurately compare the majority of the bacterial species, it would have also been infeasible based on CPU runtime. As more species and orthologs are included in the maximum likelihood analysis, the runtime increases exponentially. The fastest iteration of maximum likelihood finished in 2.5 hour on 100 mammals, using 18 orthologous genes which were each present in at least 97 species. In contrast, CAM used all genes in 107 mammals and finished in 0.2101 hours (12 minutes, 36 seconds). The slowest iteration of maximum likelihood finished in 199.75 hours on 58 fungi using 648 orthologs, which were each annotated in at least five species. CAM again analyzed all genes, both annotated and unannotated, across 234 fungi, finishing in 0.2167 hours (13 minutes).

### Ortholog frequency for maximum likelihood analysis

Maximum likelihood is highly dependent on the number of orthologs annotated in the analysis. In Table 6, we report the minimum number of species with an ortholog annotation, the number of orthologs used, and the total number of characters in the super-matrix for each taxonomic group. All orthologous genes with gene annotations spanning at least the number of species noted in column 2 (minimum number of species with orthologs) were included in the analysis. Differences in the minimum number of species with an ortholog are due to differences in the breadth of gene annotations within a taxonomic group. For instance, few orthologous gene annotations spanned more than five species in fungi, invertebrates, and protozoa; however, many orthologs were annotated in 100 vertebrate species. We did not filter the orthologs on any metric besides the number of species with that gene annotation.

**Table 6:** Matrix statistics for maximum likelihood analysis. The first column is the taxonomic group. The second column is the minimum number of species that must include an ortholog annotation for it to be included in the matrix. The third column is the number of orthologs with the minimum number of species annotations. The fourth column is the number of nucleotide characters in the combined alignment of all orthologs included in the analysis.

Taxonomic Group	Minimum number of species with ortholog	Number of orthologs in super-matrix	Characters in super-matrix
Archaea	95	45	62,442
Fungi	5	648	1,403,618
Invertebrates	5	20	17,665
Plants	40	75	87,764
Protozoa	5	200	411,028
Mammals	97	18	24,767
Other vertebrates	108	28	30,900

## Discussion

The advent of Next Generation Sequencing (NGS) enables researchers to quickly and inexpensively sequence genomes faster than orthologous relationships and species phylogenies can be annotated and examined. Therefore, alignment-free algorithms are becoming increasingly more important in determining phylogenetic trees in a cost-effective and time-efficient manner. The results of our CAM analyses show that CAM produces comparable trees to other alignment-free algorithms, performs quickly, and has the ability to compare vastly divergent species.

### CAM accuracy

Although alignment-free methods are not currently considered as accurate as alignment-based methods, as more alignment-free methods and phylogenetically conserved characters are discovered and combined, their accuracy increases. We recognize that the OTL and the NCBI reference trees suffer from biases based on the phylogenetic tree reconstruction methods used to create them. However, they provide researchers with the most comprehensive number of species by combining the results of various studies. Therefore, similarity to the reference trees is a relative metric that can be used to assess each algorithm against the results from all other algorithms. Furthermore, all algorithms are subject to the same potential biases that exist by performing this type of analysis because they are each compared to the same reference phylogenies.

CAM recovered trees that were 60–82% similar to the OTL and 70–91% similar to the NCBI Taxonomy. Although CAM does not recover identical phylogenies to the OTL or the NCBI taxonomy, the recovered phylogenies have comparable percent branch similarities as phylogenies recovered using traditional ortholog-based maximum likelihood estimates. For protozoa, the percent similarity with the OTL and the NCBI taxonomy was only 1% different between maximum likelihood and CAM. Species relationships recovered for archaea, mammals, and other vertebrates were more similar to established phylogenies using maximum likelihood. However, since traditional ortholog-based techniques were used to construct the current representation of the OTL, it is expected that taxonomic groups with well-documented orthologs should recover very similar trees to the reference. CAM recovered trees were comparable in percent similarity to other alignment-free algorithms. No single algorithm outperformed all other algorithms in terms of percent similarity with the OTL or the NCBI taxonomy. Since CAM performed comparably to all other alignment-free algorithms, codon aversion motifs should be considered in conjunction with these other methods in phylogenomic analyses.

Amino acid aversion motifs also recovered trees that were comparable to the OTL and NCBI taxonomy. Since amino acid aversion recovered trees with similar percent identities as the other alignment-free algorithms, amino acids might be sufficient to determine phylogenetic relationships when only protein sequences are available. However, CAM performed slightly better than amino acid aversion in the majority of the analyses, indicating that codon aversion provides additional phylogenetic information. This difference may be due to the larger number of possible codon aversion motifs (2^61^) as opposed to amino acid aversion motifs (2^20^). This additional information allows CAM to distinguish the relationships between species at a higher resolution in the majority of analyses, indicating that codon aversion provides additional phylogenetic information.

We considered the possibility that gene lengths influence CAM’s algorithm. Since fewer codons are present in short genes, there are potentially more codons that are avoided by random chance. This potential bias could cause genomes with a preponderance of short genes to be clustered based on gene size as opposed to a codon or amino acid bias within the gene. To determine if this bias affected our analysis, we analyzed the frequency of the number of codons excluded in each codon aversion motif (Figs. S22–S31). If short gene bias were prevalent, we would expect to observe an evenly distributed number of codons in each codon aversion motif, ranging from two to about sixty (indicating that long genes used all available codons and short genes used few available codons). We graphed these frequencies and determined that each of the taxonomic groups showed the same trend of codon aversion motifs. On average, relatively few codons were included in each motif (14.4819 codons with a standard deviation of 8.6881).

CAM is also robust to partial gene annotations. Including or excluding partial gene sequences in the analysis had a minimal effect on the overall species relationships. This analysis indicates that missing data or incomplete data has a minimal effect on the algorithm. Furthermore, without relying on gene alignments, the recovered phylogeny is not dependent on the accuracy of the aligner or ortholog annotations. This property of all alignment-free algorithms facilitates a more universal technique to compare distantly related species that might have incorrectly labeled genes or highly mutated orthologs.

### CAM runtime

Although CAM requires genomes to be assembled with CDS regions annotated, it does not require an alignment of the genes against other species, nor does it require the time-consuming approaches of traditional methods such as maximum likelihood. Codon aversion motifs provide a basis for alignment-free methods to recover robust phylogenies quickly and with sufficient resolution to account for future species discovery. In contrast to maximum likelihood, most cladal relationships were recovered using CAM within minutes. CAM had comparable runtimes to FFP and CVTree, and faster runtimes by several orders of magnitude than some of the alignment-free methods, including ACS, Andi, and FSWM. Therefore, we show that CAM is a time-efficient alignment-free method that is comparable or faster than other alignment-free algorithms.

### CAM applies to more species than maximum likelihood

Since alignment-free methods, such as CAM, are not dependent on ortholog annotations, they are able to recover species relationships when gene sequences lack ortholog annotations. For example, ortholog annotations in protozoa were sufficient for only 24 species, whereas CAM recovered 75 taxonomic relationships. Maximum likelihood recovered only 58 species relationships for fungi, whereas CAM recovered 234 relationships. Since ortholog annotations are a limiting factor in phylogenomic studies, alignment-free methods provide the ability to recover a higher number of species relationships than traditional techniques.

CAM consistently recovers comparable phylogenies compared with other alignment-free techniques. Since CAM uses a single character state, codon aversion, across all domains of life, it limits *ad hoc* hypotheses by facilitating a single analysis of all species instead of piecing together the phylogenetic signal from different genes. Additionally, codon aversion motifs can be used to examine coevolutionary forces between different domains, such as viruses and hosts. Since similarities in codon usages have previously been identified between some viruses and their respective hosts (Chantawannakul & Cutler, 2008; Miller et al., 2017b), this technique could facilitate coevolutionary analyses by identifying overlapping motifs in distantly related species, which can then be analyzed using traditional techniques.

## Conclusions

We understand that certain limitations to our study exist. For instance, while we have shown that CAM successfully recovers most species relationships with similar accuracy as other alignment-free methods, we do not fully understand the biological mechanisms that govern the phylogenetic signal we identified. One potential explanation is that codon aversion is conserved due to selection on translational efficiency. A limited supply of tRNA exist in a given organism, and codons that do not directly complement all three anti-codons in the tRNA are generally considered suboptimal. Although suboptimal codons are sometimes preferred (Tuller et al., 2010), they generally slow translation and decrease gene expression (Quax et al., 2015).

The phylogenetic signal could also be attributed to neutral processes such as GC biased gene conversion, since GC content changes during meiosis and is therefore likely to vary directly with evolutionary time. We also note that alignment-free methods often appear as a “black box” to researchers who are accustomed to homologous character analyses that allow for directly identifying nucleotide differences in sequences. While CAM presents a paradigm shift, it has the potential to be as informative as analyses of homologous character states. Since CAM is based in codon usages within each gene, we propose that percent similarities in codon aversions between species represents similarities in the mechanisms that maintain these codon usages. Although these mechanisms are presently not fully understood, we show that they are phylogenetically conserved and can be utilized to recover a phylogeny using our method.

##  Supplemental Information

10.7717/peerj.6984/supp-1 Supplemental Information 1 Supplemental Note 1: Summary of CAM Options A short summary of available parameters to modify the output from CAM.

10.7717/peerj.6984/supp-2 Table S1 The advantages and disadvantages of each phylogenetic comparison metric The first column is the name of the metric. The second column is a short description of how the metric works. The third and fourth columns explain the advantages and disadvantages of each method, respectively.

10.7717/peerj.6984/supp-3 Figure S1 Frequency of Partial Genes This figure shows the proportion of partial genes in each clade. A partial gene is defined as a gene in which we do not have the entire DNA sequence available. Each boxplot represents the distribution of the proportion of partial genes in each species of the clade.

10.7717/peerj.6984/supp-4 Figure S2 All Clades - Motifs Found in Multiple Species vs. Unique Motifs Shows how many motifs are shared in different genes within the same clade versus how many motifs are unique to a single gene.

10.7717/peerj.6984/supp-5 Figure S3 Archaea - Motifs Found in Multiple Species vs. Unique Motifs Shows how many motifs are shared in different genes within the same clade versus how many motifs are unique to a single gene.

10.7717/peerj.6984/supp-6 Figure S4 Bacteria - Motifs Found in Multiple Species vs. Unique Motifs Shows how many motifs are shared in different genes within the same clade versus how many motifs are unique to a single gene.

10.7717/peerj.6984/supp-7 Figure S5 Fungi - Motifs Found in Multiple Species vs. Unique Motifs Shows how many motifs are shared in different genes within the same clade versus how many motifs are unique to a single gene.

10.7717/peerj.6984/supp-8 Figure S6 Invertebrates - Motifs Found in Multiple Species vs. Unique Motifs Shows how many motifs are shared in different genes within the same clade versus how many motifs are unique to a single gene.

10.7717/peerj.6984/supp-9 Figure S7 Mammals - Motifs Found in Multiple Species vs. Unique Motifs Shows how many motifs are shared in different genes within the same clade versus how many motifs are unique to a single gene.

10.7717/peerj.6984/supp-10 Figure S8 Plants - Motifs Found in Multiple Species vs. Unique Motifs Shows how many motifs are shared in different genes within the same clade versus how many motifs are unique to a single gene.

10.7717/peerj.6984/supp-11 Figure S9 Protozoa - Motifs Found in Multiple Species vs. Unique Motifs Shows how many motifs are shared in different genes within the same clade versus how many motifs are unique to a single gene.

10.7717/peerj.6984/supp-12 Figure S10 Vertebrate Other - Motifs Found in Multiple Species vs. Unique Motifs Shows how many motifs are shared in different genes within the same clade versus how many motifs are unique to a single gene.

10.7717/peerj.6984/supp-13 Figure S11 Viruses - Motifs Found in Multiple Species vs. Unique Motifs Shows how many motifs are shared in different genes within the same clade versus how many motifs are unique to a single gene.

10.7717/peerj.6984/supp-14 Figure S12 All Clades - Frequency of Codon Aversion by Codon The frequency of codon exclusion for the taxonomic group. The box plot represents the frequency of species in the taxonomic group that exclude a certain codon in their genes (e.g., if a codon is not used in 50% of a species’ genes, then that species would be plotted at 0.50).

10.7717/peerj.6984/supp-15 Figure S13 Archaea - Frequency of Codon Aversion by Codon The frequency of codon exclusion for the taxonomic group. The box plot represents the frequency of species in the taxonomic group that exclude a certain codon in their genes (e.g., if a codon is not used in 50% of a species’ genes, then that species would be plotted at 0.50).

10.7717/peerj.6984/supp-16 Figure S14 Bacteria - Frequency of Codon Aversion by Codon The frequency of codon exclusion for the taxonomic group. The box plot represents the frequency of species in the taxonomic group that exclude a certain codon in their genes (e.g., if a codon is not used in 50% of a species’ genes, then that species would be plotted at 0.50).

10.7717/peerj.6984/supp-17 Figure S15 Fungi - Frequency of Codon Aversion by Codon The frequency of codon exclusion for the taxonomic group. The box plot represents the frequency of species in the taxonomic group that exclude a certain codon in their genes (e.g., if a codon is not used in 50% of a species’ genes, then that species would be plotted at 0.50).

10.7717/peerj.6984/supp-18 Figure S16 Invertebrates - Frequency of Codon Aversion by Codon The frequency of codon exclusion for the taxonomic group. The box plot represents the frequency of species in the taxonomic group that exclude a certain codon in their genes (e.g., if a codon is not used in 50% of a species’ genes, then that species would be plotted at 0.50).

10.7717/peerj.6984/supp-19 Figure S17 Mammals - Frequency of Codon Aversion by Codon The frequency of codon exclusion for the taxonomic group. The box plot represents the frequency of species in the taxonomic group that exclude a certain codon in their genes (e.g., if a codon is not used in 50% of a species’ genes, then that species would be plotted at 0.50).

10.7717/peerj.6984/supp-20 Figure S18 Plants - Frequency of Codon Aversion by Codon The frequency of codon exclusion for the taxonomic group. The box plot represents the frequency of species in the taxonomic group that exclude a certain codon in their genes (e.g., if a codon is not used in 50% of a species’ genes, then that species would be plotted at 0.50).

10.7717/peerj.6984/supp-21 Figure S19 Protozoa - Frequency of Codon Aversion by Codon The frequency of codon exclusion for the taxonomic group. The box plot represents the frequency of species in the taxonomic group that exclude a certain codon in their genes (e.g., if a codon is not used in 50% of a species’ genes, then that species would be plotted at 0.50).

10.7717/peerj.6984/supp-22 Figure S20 Vertebrate Other - Frequency of Codon Aversion by Codon The frequency of codon exclusion for the taxonomic group. The box plot represents the frequency of species in the taxonomic group that exclude a certain codon in their genes (e.g., if a codon is not used in 50% of a species’ genes, then that species would be plotted at 0.50).

10.7717/peerj.6984/supp-23 Figure S21 Viruses - Frequency of Codon Aversion by Codon The frequency of codon exclusion for the taxonomic group. The box plot represents the frequency of species in the taxonomic group that exclude a certain codon in their genes (e.g., if a codon is not used in 50% of a species’ genes, then that species would be plotted at 0.50).

10.7717/peerj.6984/supp-24 Figure S22 All Clades - Number of Codons Excluded in Motifs Shows the frequency of how many codons (0-64) are not used in each gene.

10.7717/peerj.6984/supp-25 Figure S23 Archaea - Number of Codons Excluded in Motifs Shows the frequency of how many codons (0-64) are not used in each gene.

10.7717/peerj.6984/supp-26 Figure S24 Bacteria - Number of Codons Excluded in Motifs Shows the frequency of how many codons (0-64) are not used in each gene.

10.7717/peerj.6984/supp-27 Figure S25 Fungi - Number of Codons Excluded in Motifs Shows the frequency of how many codons (0-64) are not used in each gene.

10.7717/peerj.6984/supp-28 Figure S26 Invertebrates - Number of Codons Excluded in Motifs Shows the frequency of how many codons (0-64) are not used in each gene.

10.7717/peerj.6984/supp-29 Figure S27 Mammals - Number of Codons Excluded in Motifs Shows the frequency of how many codons (0-64) are not used in each gene.

10.7717/peerj.6984/supp-30 Figure S28 Protozoa - Number of Codons Excluded in Motifs Shows the frequency of how many codons (0-64) are not used in each gene.

10.7717/peerj.6984/supp-31 Figure S29 Plants - Number of Codons Excluded in Motifs Shows the frequency of how many codons (0-64) are not used in each gene.

10.7717/peerj.6984/supp-32 Figure S30 Vertebrate Other - Number of Codons Excluded in Motifs Shows the frequency of how many codons (0-64) are not used in each gene.

10.7717/peerj.6984/supp-33 Figure S31 Viruses - Number of Codons Excluded in Motifs Shows the frequency of how many codons (0-64) are not used in each gene.

10.7717/peerj.6984/supp-34 Figure S32 All Clades - Repeated Motifs The frequency of which codon motifs are repeated is shown. The x- axis depicts how many time a motif was repeated in all the genes in a clade. The y-axis depicts how many motifs were repeated a given number of times (shown in the natural log). Some outliers were removed from each graph for clarity. These outliers represent the motifs in which only stop codons are excluded. All clades outliers excluded: (1309911,1), (2185083,1), (2433089,1).

10.7717/peerj.6984/supp-35 Figure S33 Archaea - Repeated Motifs The frequency of which codon motifs are repeated is shown. The x- axis depicts how many time a motif was repeated in all the genes in a clade. The y-axis depicts how many motifs were repeated a given number of times (shown in the natural log). Some outliers were removed from each graph for clarity. These outliers represent the motifs in which only stop codons are excluded. Archaea outliers excluded: (10360,1), (10564,1).

10.7717/peerj.6984/supp-36 Figure S34 Bacteria - Repeated Motifs The frequency of which codon motifs are repeated is shown. The x- axis depicts how many time a motif was repeated in all the genes in a clade. The y-axis depicts how many motifs were repeated a given number of times (shown in the natural log). Some outliers were removed from each graph for clarity. These outliers represent the motifs in which only stop codons are excluded. Bacteria outliers excluded: (681998,1), (1085854,1), (1611727,1).

10.7717/peerj.6984/supp-37 Figure S37 Fungi - Repeated Motifs The frequency of which codon motifs are repeated is shown. The x- axis depicts how many time a motif was repeated in all the genes in a clade. The y-axis depicts how many motifs were repeated a given number of times (shown in the natural log). Some outliers were removed from each graph for clarity. These outliers represent the motifs in which only stop codons are excluded. Fungi outliers excluded: (140907,0), (157884,1), (226451,1).

10.7717/peerj.6984/supp-38 Figure S36 Invertebrates - Repeated Motifs The frequency of which codon motifs are repeated is shown. The x- axis depicts how many time a motif was repeated in all the genes in a clade. The y-axis depicts how many motifs were repeated a given number of times (shown in the natural log). Some outliers were removed from each graph for clarity. These outliers represent the motifs in which only stop codons are excluded. Invertebrates outliers excluded: (110662,1), (201864,1), (166597,1).

10.7717/peerj.6984/supp-39 Figure S37 Mammals - Repeated Motifs The frequency of which codon motifs are repeated is shown. The x- axis depicts how many time a motif was repeated in all the genes in a clade. The y-axis depicts how many motifs were repeated a given number of times (shown in the natural log). Some outliers were removed from each graph for clarity. These outliers represent the motifs in which only stop codons are excluded. Mammal outliers excluded: (81051,1), (105156,1), (17812,1).

10.7717/peerj.6984/supp-40 Figure S38 Plants - Repeated Motifs The frequency of which codon motifs are repeated is shown. The x- axis depicts how many time a motif was repeated in all the genes in a clade. The y-axis depicts how many motifs were repeated a given number of times (shown in the natural log). Some outliers were removed from each graph for clarity. These outliers represent the motifs in which only stop codons are excluded. Plants outliers excluded: (158430,1), (127795,1), (224688,1).

10.7717/peerj.6984/supp-41 Figure S39 Protozoa - Repeated Motifs The frequency of which codon motifs are repeated is shown. The x- axis depicts how many time a motif was repeated in all the genes in a clade. The y-axis depicts how many motifs were repeated a given number of times (shown in the natural log). Some outliers were removed from each graph for clarity. These outliers represent the motifs in which only stop codons are excluded. Protozoa Outliers excluded: (32139,1), (41048,1), (30539,1).

10.7717/peerj.6984/supp-42 Figure S40 Vertebrate Other - Repeated Motifs The frequency of which codon motifs are repeated is shown. The x- axis depicts how many time a motif was repeated in all the genes in a clade. The y-axis depicts how many motifs were repeated a given number of times (shown in the natural log). Some outliers were removed from each graph for clarity. These outliers represent the motifs in which only stop codons are excluded. Vertebrate other outliers excluded: (167892,1), (114746,1), (254804,1).

10.7717/peerj.6984/supp-43 Figure S41 Viruses - Repeated Motifs The frequency of which codon motifs are repeated is shown. The x- axis depicts how many time a motif was repeated in all the genes in a clade. The y-axis depicts how many motifs were repeated a given number of times (shown in the natural log). Some outliers were removed from each graph for clarity. These outliers represent the motifs in which only stop codons are excluded. Virus outliers excluded: (2669,1), (2167,1), (4664,1)

## Additional Information and Declarations

### Data Availability

The following information was supplied regarding data availability: CAM is available at https://github.com/ridgelab/cam. All genomes used in our analyses were downloaded from the RefSeq FTP site at ftp://ftp.ncbi.nlm.nih.gov/genomes/refseq/.
