# Supplementary material for: CAM: an alignment-free method to recover phylogenies using codon aversion motifs
Source: PeerJ. 2019 Jun 4;7:e6984. doi: 10.7717/peerj.6984 (PMC6555396; doi:10.7717/peerj.6984)
Supplement: Supplemental Information 1 — A short summary of available parameters to modify the output from CAM. [file peerj-07-6984-s001.docx]

Supplementary Note 1

**Summary of CAM Options**

Several additional options are available for cam.py that allow users greater flexibility to run CAM and recover a distance matrix based on their preferences. An input FASTA file must be provided either using a list (standard bash expansion) through the -i option, or by providing the name of a directory through the -id option. Compressed files (gzip) are accepted and automatically handled with the .gz file extension. By default, all processing cores are used by CAM, although any number of cores can be specified by using the -t option. By default, the output is written to standard out, although an output file path can be supplied by using the -o option. If memory constraints are an issue, the distance matrix can be calculated species-by-species through the -w option; however, the header line will be written at the end of the file instead of the beginning if this option is used. By default, DNA sequences are expected by CAM. For convenience, we also provide the -rna flag if the FASTA files are RNA sequences and the -a flag if they are protein sequences. If the user desires to run amino acid motifs from DNA or RNA sequences, we also provide the -aa option which translates DNA or RNA (if the -rna flag is set) to amino acids. Finally, by default species must share at least 5% of their usage motifs to not be given the maximum distance (1.0). This option can be modified using the -p option, although it is not recommended to change this option if the species have few genes because the 5% threshold prevents false positives from small genome bias.
