# Supplementary material for: CAM: an alignment-free method to recover phylogenies using codon aversion motifs
Source: PeerJ. 2019 Jun 4;7:e6984. doi: 10.7717/peerj.6984 (PMC6555396; doi:10.7717/peerj.6984)
Supplement: Table S1 — The first column is the name of the metric. The second column is a short description of how the metric works. The third and fourth columns explain the advantages and disadvantages of each method, respectively. [file peerj-07-6984-s002.docx]

Supplementary Table S1

| Metric | Description | Advantages | Shortcomings |
| --- | --- | --- | --- |
| Percentage of edge similarity | Percentage of branches in both query and subject trees | - Useful for large trees  - Useful for trees that contain polytomies | - Does not provide specific information of the location of differences. |
| Robinson Foulds Distance | Counts number of edges present in one tree, but not the other tree | - Independent from any model of tree editing  - Relies only on current characteristics of the two trees | - Sensitive to small changes in leaf nodes  - Provides low amount of discrimination for large trees |
| Maximum Agreement SubTree | Determines the smallest collection of leaves that, when removed, induce the same tree | - Useful for sizeable collections of trees  - Useful for smaller trees with “rogue” taxa (taxa whose placement is unclear) | - Requiring exact agreement is computationally demanding and may lead to inaccurate results |
| Edit distance metrics (Nearest Neighbor Interchange, Subtree Prune and Regraft, Tree Bisection and Reconnection) | Smallest number of allowed operations that will transform one tree into another | - Useful for smaller trees  - Useful for when the change operations done on trees are known | - NP-hard  - Unclear which operations to use |
