# Supplementary figures and images for: CAM: an alignment-free method to recover phylogenies using codon aversion motifs
Source: PeerJ. 2019 Jun 4;7:e6984. doi: 10.7717/peerj.6984 (PMC6555396; doi:10.7717/peerj.6984)

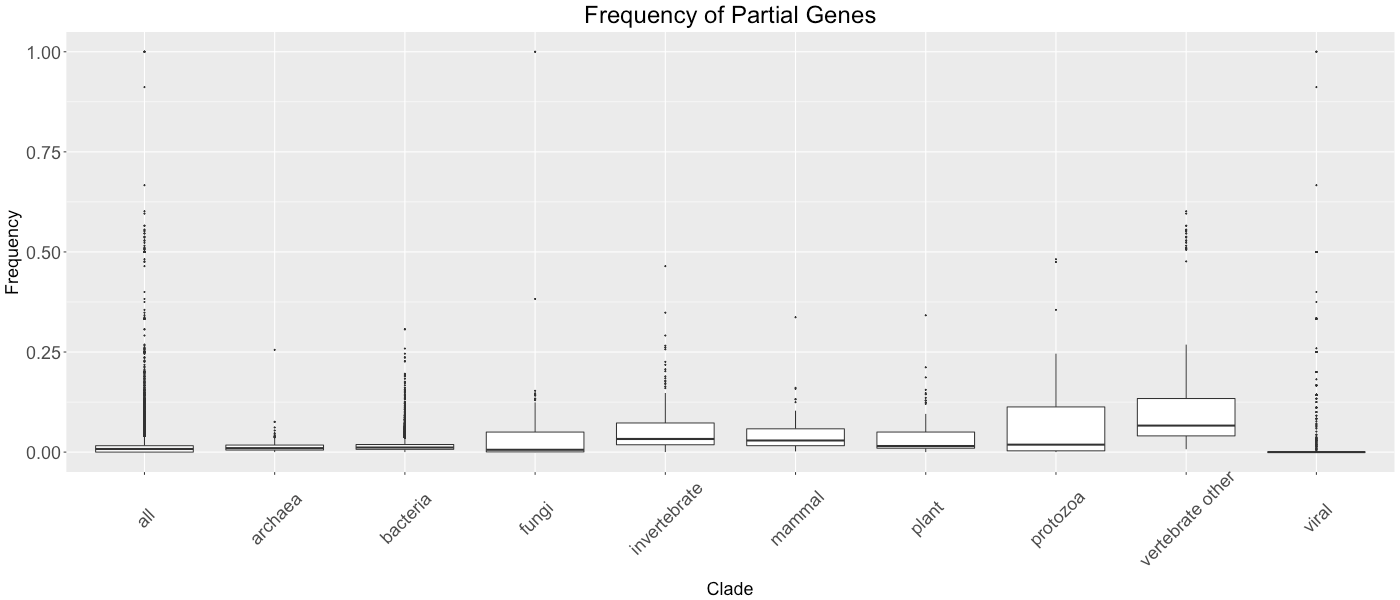

Supplement: Figure S1 — This figure shows the proportion of partial genes in each clade. A partial gene is defined as a gene in which we do not have the entire DNA sequence available. Each boxplot represents the distribution of the proportion of partial genes in each species of the clade. [file peerj-07-6984-s003.png]

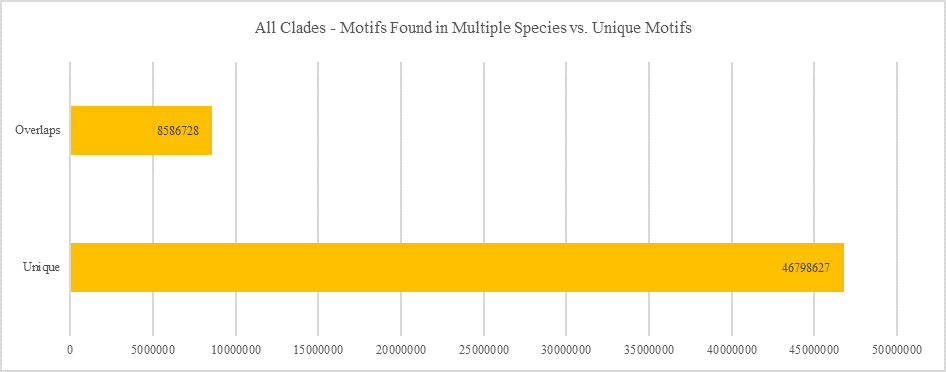

Supplement: Figure S2 — Shows how many motifs are shared in different genes within the same clade versus how many motifs are unique to a single gene. [file peerj-07-6984-s004.png]

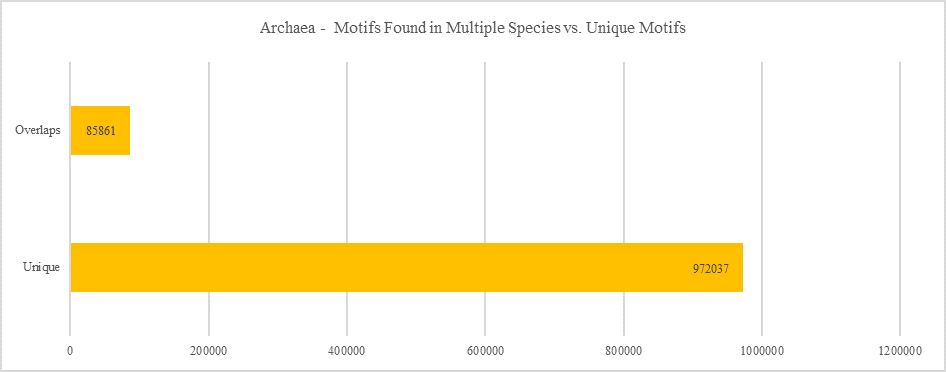

Supplement: Figure S3 — Shows how many motifs are shared in different genes within the same clade versus how many motifs are unique to a single gene. [file peerj-07-6984-s005.png]

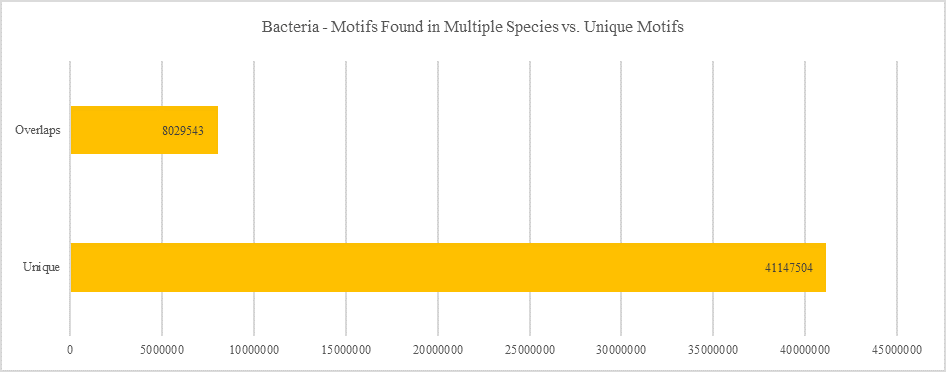

Supplement: Figure S4 — Shows how many motifs are shared in different genes within the same clade versus how many motifs are unique to a single gene. [file peerj-07-6984-s006.png]

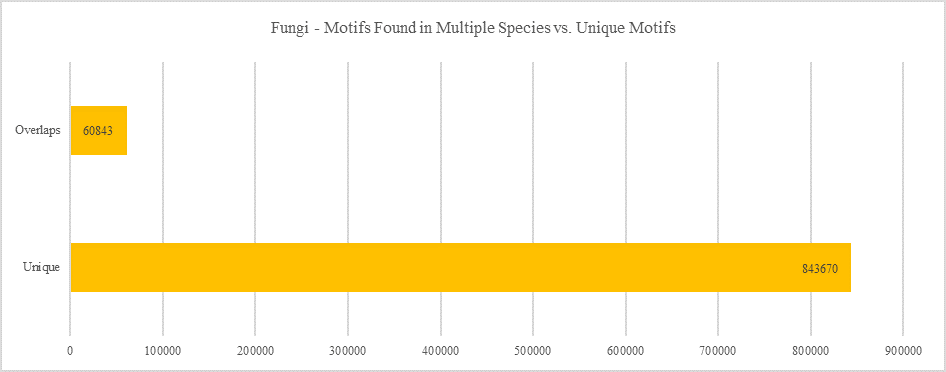

Supplement: Figure S5 — Shows how many motifs are shared in different genes within the same clade versus how many motifs are unique to a single gene. [file peerj-07-6984-s007.png]

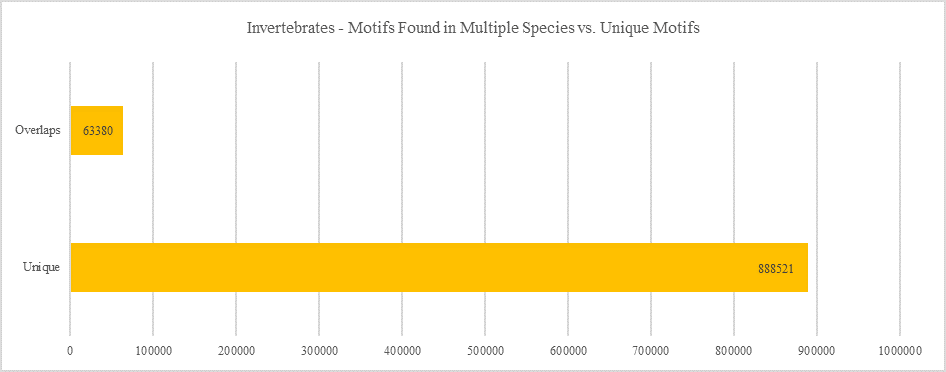

Supplement: Figure S6 — Shows how many motifs are shared in different genes within the same clade versus how many motifs are unique to a single gene. [file peerj-07-6984-s008.png]

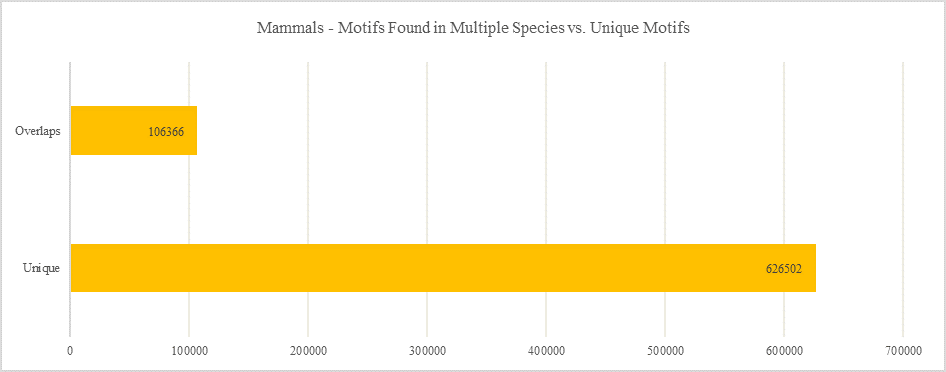

Supplement: Figure S7 — Shows how many motifs are shared in different genes within the same clade versus how many motifs are unique to a single gene. [file peerj-07-6984-s009.png]

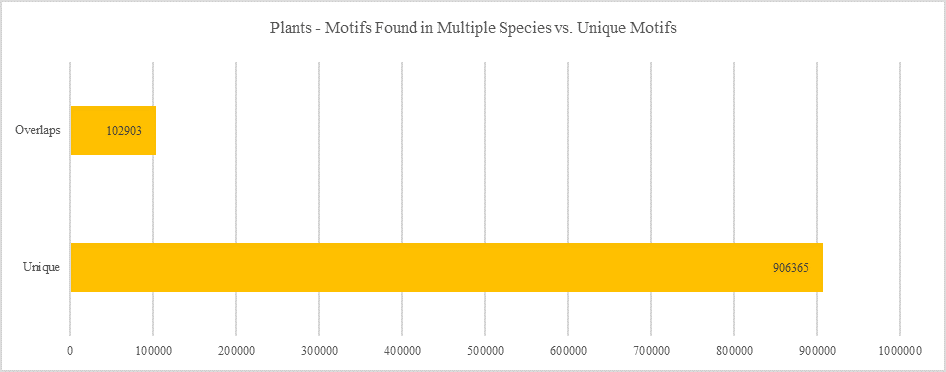

Supplement: Figure S8 — Shows how many motifs are shared in different genes within the same clade versus how many motifs are unique to a single gene. [file peerj-07-6984-s010.png]

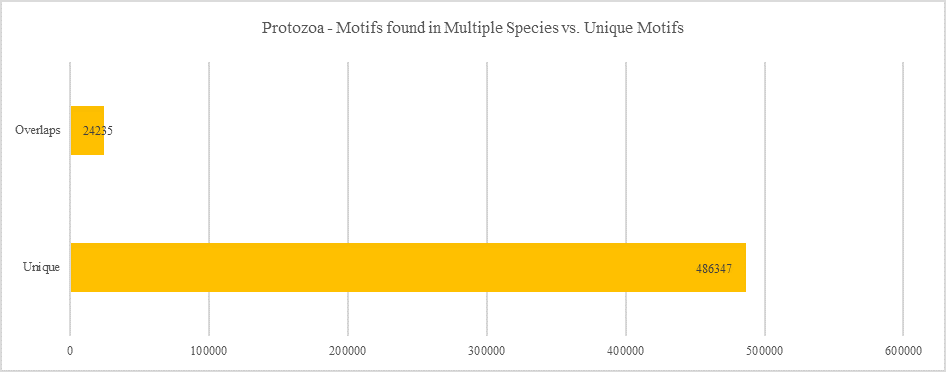

Supplement: Figure S9 — Shows how many motifs are shared in different genes within the same clade versus how many motifs are unique to a single gene. [file peerj-07-6984-s011.png]

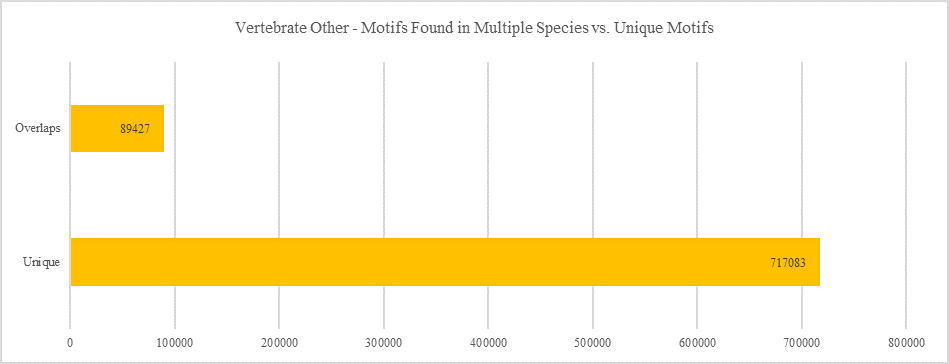

Supplement: Figure S10 — Shows how many motifs are shared in different genes within the same clade versus how many motifs are unique to a single gene. [file peerj-07-6984-s012.png]

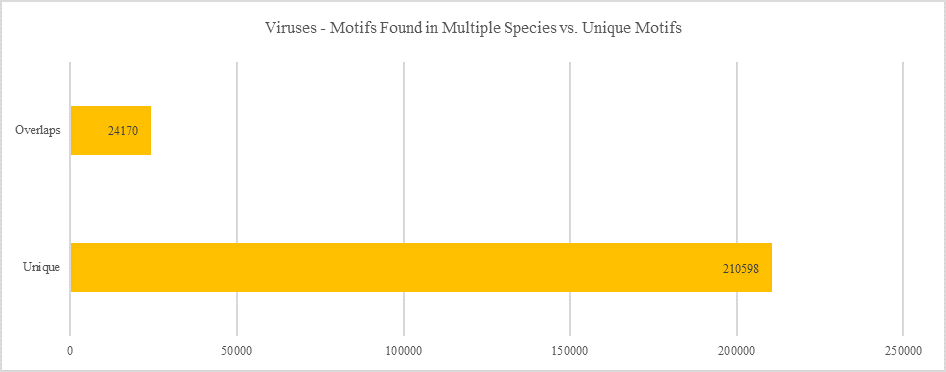

Supplement: Figure S11 — Shows how many motifs are shared in different genes within the same clade versus how many motifs are unique to a single gene. [file peerj-07-6984-s013.png]

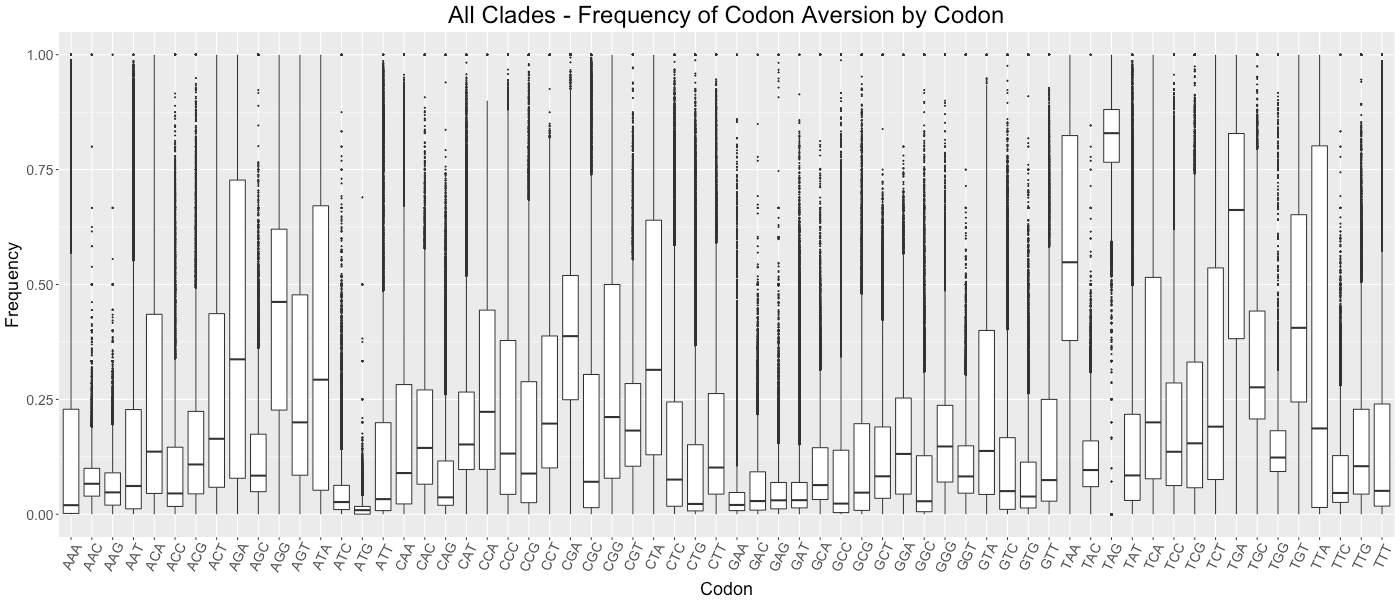

Supplement: Figure S12 — The frequency of codon exclusion for the taxonomic group. The box plot represents the frequency of species in the taxonomic group that exclude a certain codon in their genes (e.g., if a codon is not used in 50% of a species’ genes, then that species would be plotted at 0.50). [file peerj-07-6984-s014.png]

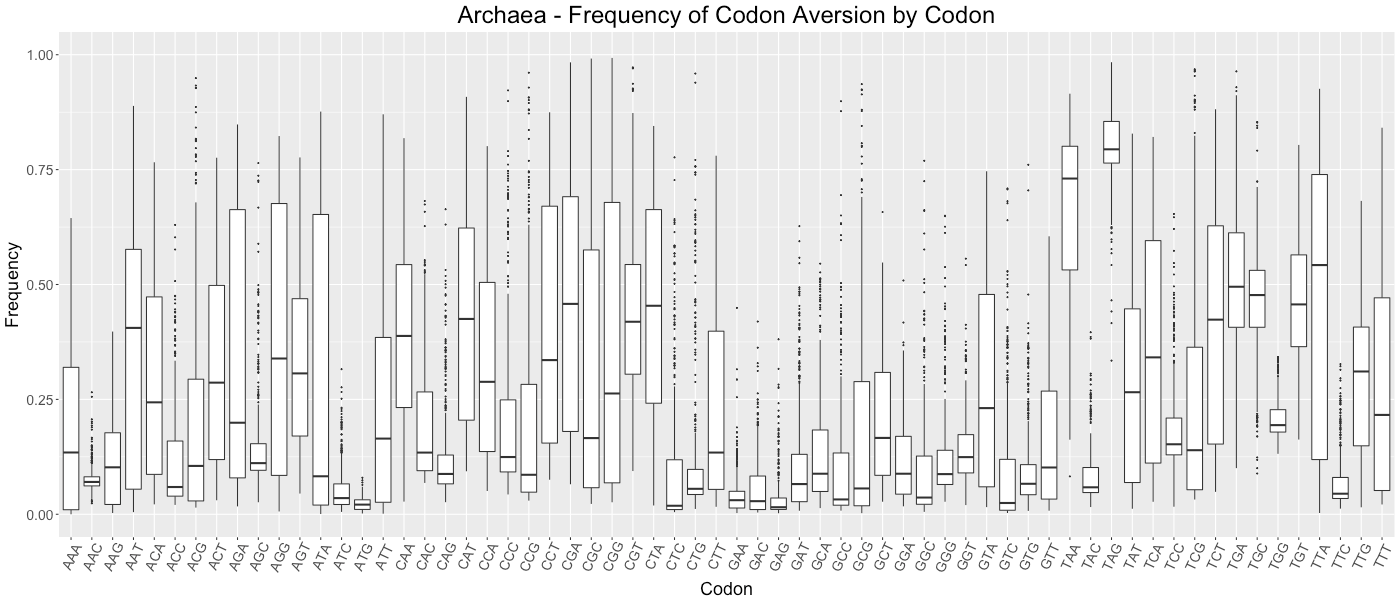

Supplement: Figure S13 — The frequency of codon exclusion for the taxonomic group. The box plot represents the frequency of species in the taxonomic group that exclude a certain codon in their genes (e.g., if a codon is not used in 50% of a species’ genes, then that species would be plotted at 0.50). [file peerj-07-6984-s015.png]

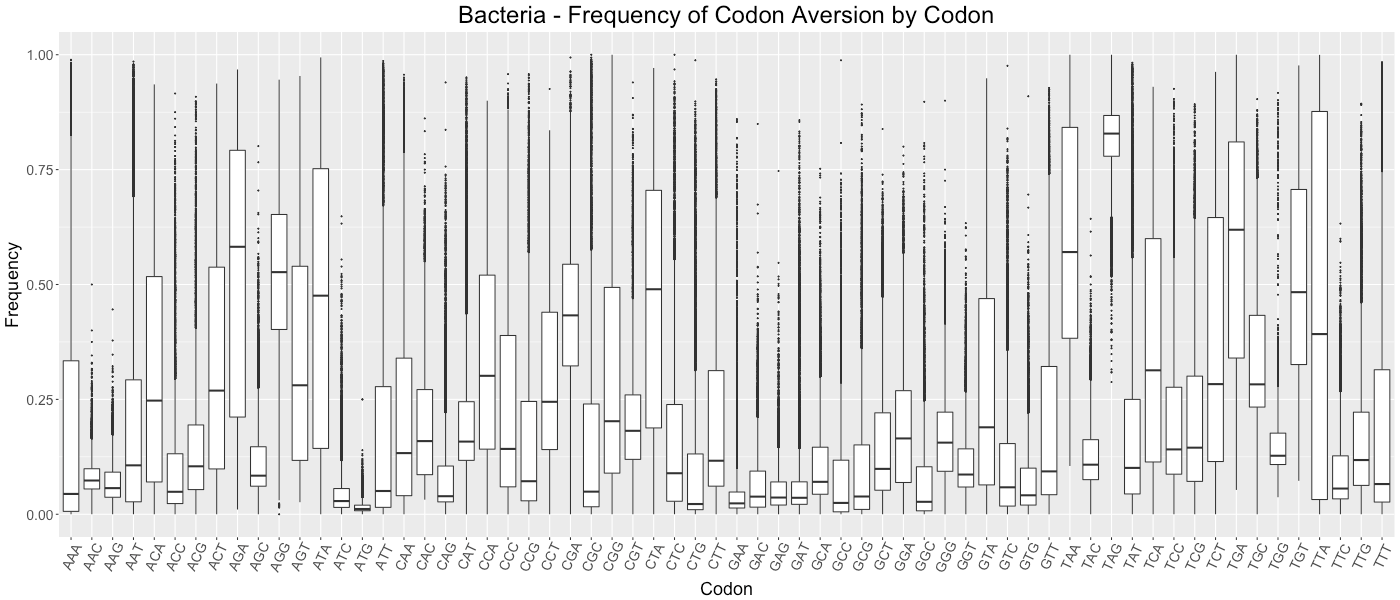

Supplement: Figure S14 — The frequency of codon exclusion for the taxonomic group. The box plot represents the frequency of species in the taxonomic group that exclude a certain codon in their genes (e.g., if a codon is not used in 50% of a species’ genes, then that species would be plotted at 0.50). [file peerj-07-6984-s016.png]

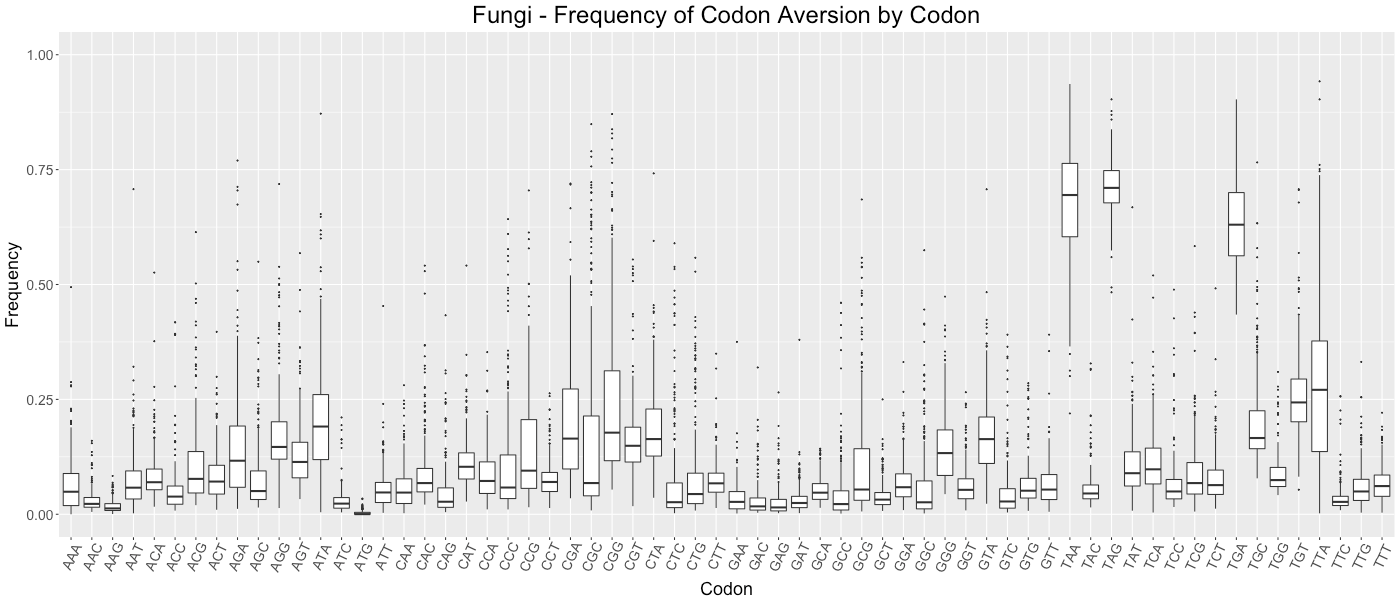

Supplement: Figure S15 — The frequency of codon exclusion for the taxonomic group. The box plot represents the frequency of species in the taxonomic group that exclude a certain codon in their genes (e.g., if a codon is not used in 50% of a species’ genes, then that species would be plotted at 0.50). [file peerj-07-6984-s017.png]

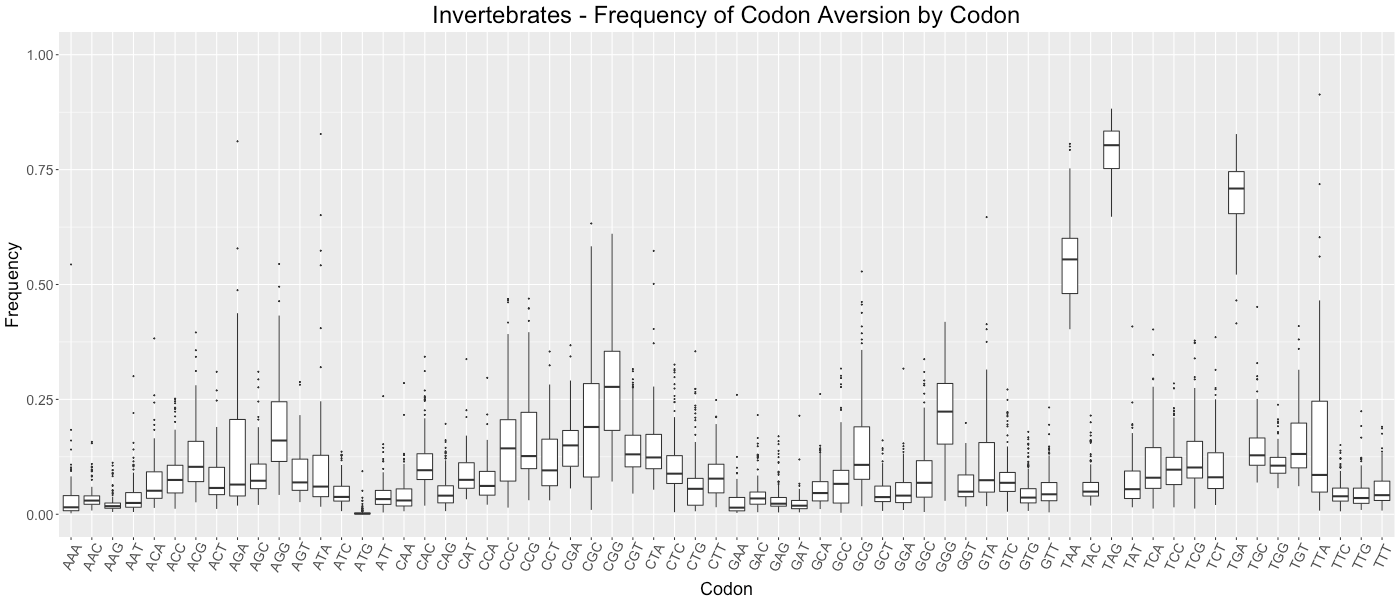

Supplement: Figure S16 — The frequency of codon exclusion for the taxonomic group. The box plot represents the frequency of species in the taxonomic group that exclude a certain codon in their genes (e.g., if a codon is not used in 50% of a species’ genes, then that species would be plotted at 0.50). [file peerj-07-6984-s018.png]

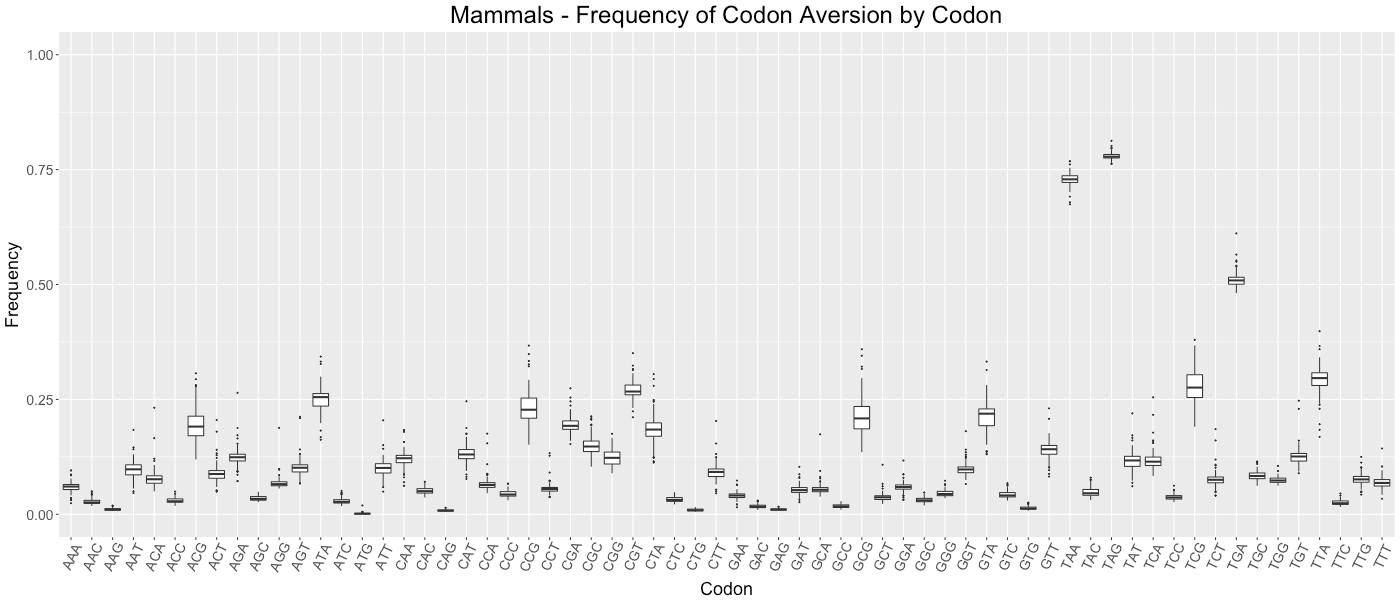

Supplement: Figure S17 — The frequency of codon exclusion for the taxonomic group. The box plot represents the frequency of species in the taxonomic group that exclude a certain codon in their genes (e.g., if a codon is not used in 50% of a species’ genes, then that species would be plotted at 0.50). [file peerj-07-6984-s019.png]

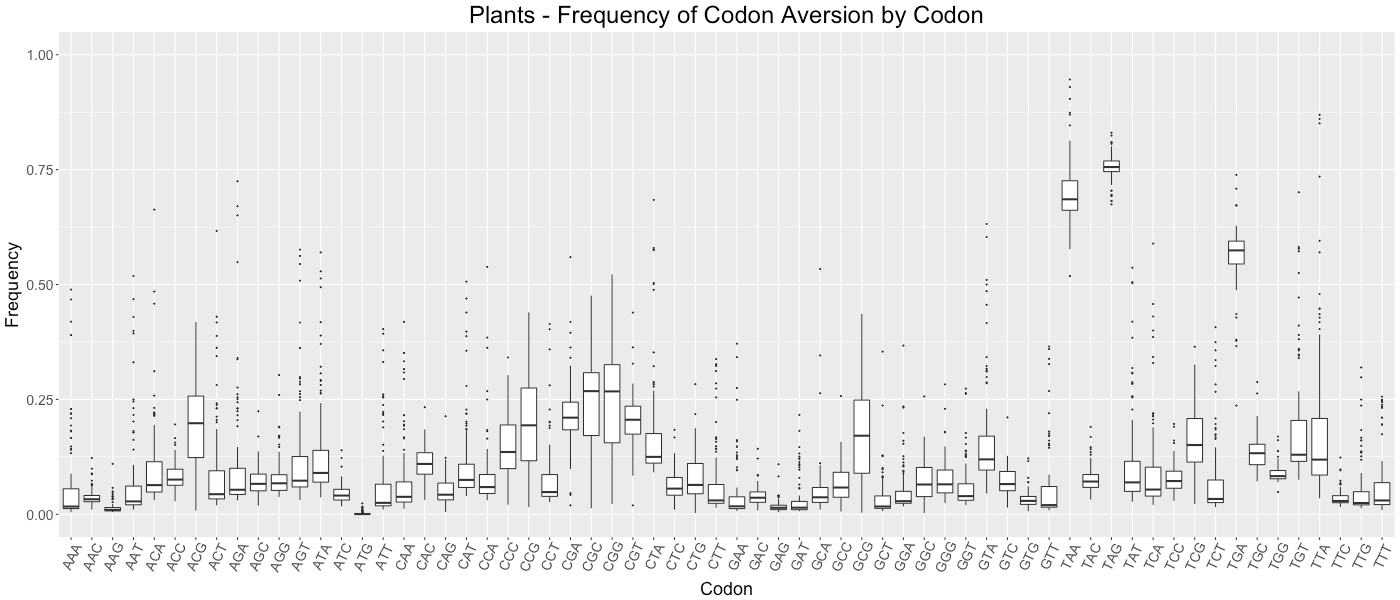

Supplement: Figure S18 — The frequency of codon exclusion for the taxonomic group. The box plot represents the frequency of species in the taxonomic group that exclude a certain codon in their genes (e.g., if a codon is not used in 50% of a species’ genes, then that species would be plotted at 0.50). [file peerj-07-6984-s020.png]

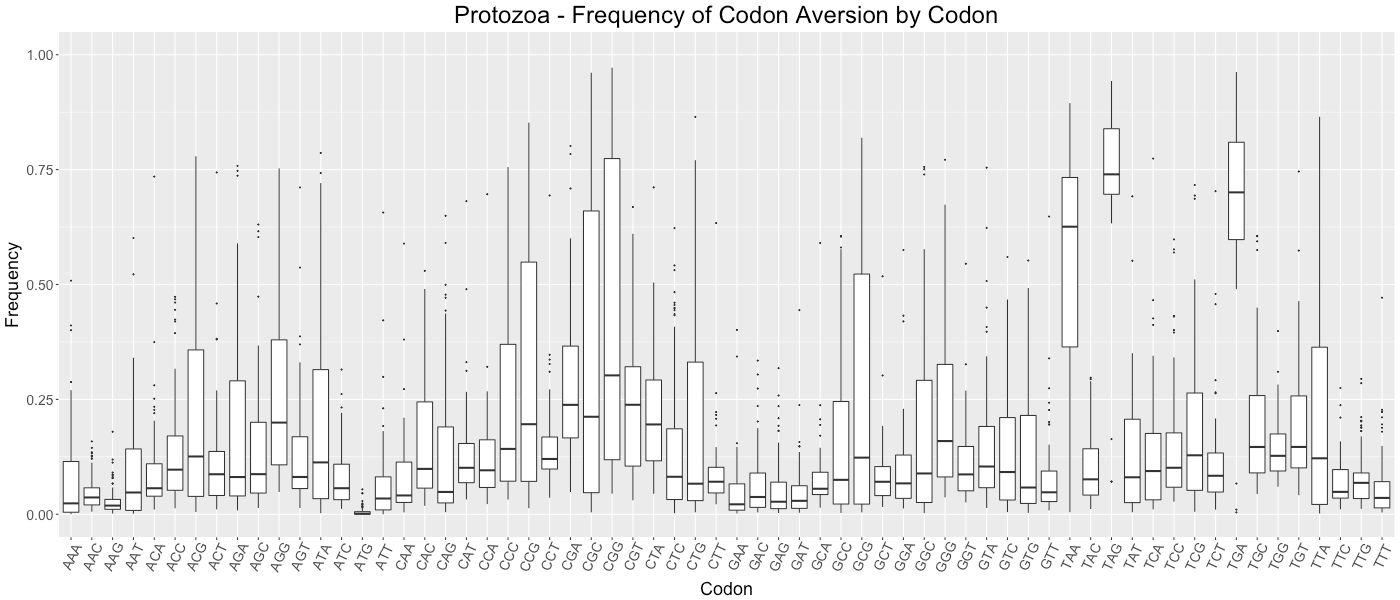

Supplement: Figure S19 — The frequency of codon exclusion for the taxonomic group. The box plot represents the frequency of species in the taxonomic group that exclude a certain codon in their genes (e.g., if a codon is not used in 50% of a species’ genes, then that species would be plotted at 0.50). [file peerj-07-6984-s021.png]

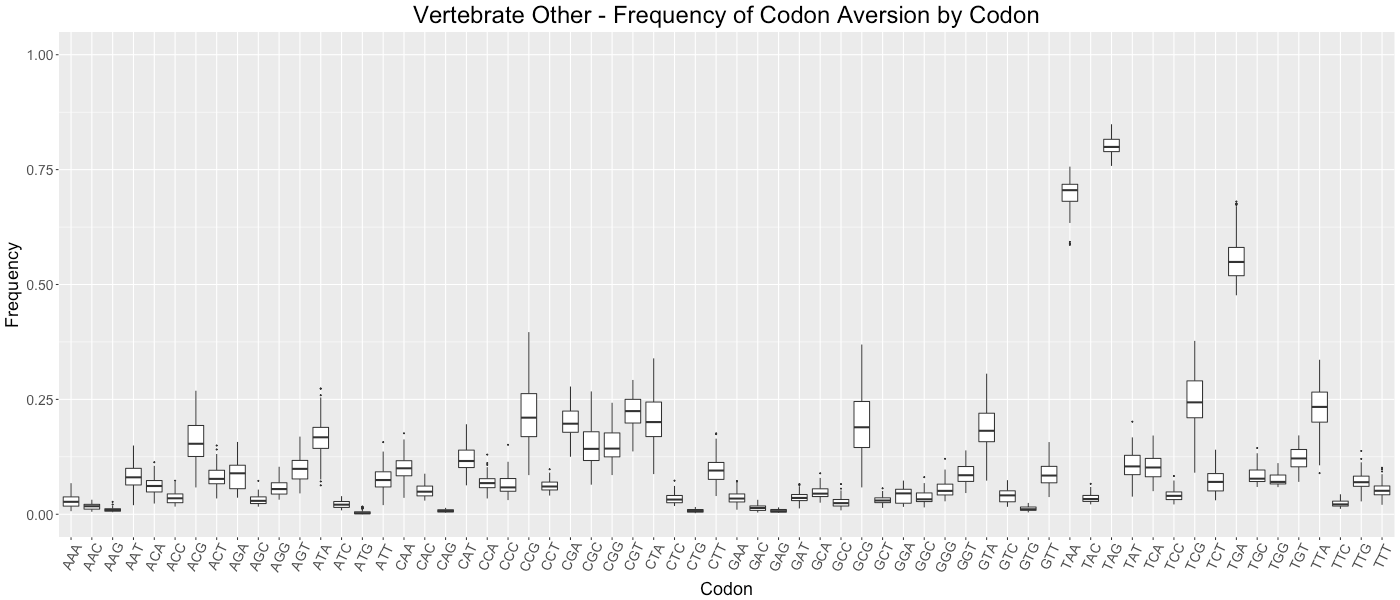

Supplement: Figure S20 — The frequency of codon exclusion for the taxonomic group. The box plot represents the frequency of species in the taxonomic group that exclude a certain codon in their genes (e.g., if a codon is not used in 50% of a species’ genes, then that species would be plotted at 0.50). [file peerj-07-6984-s022.png]

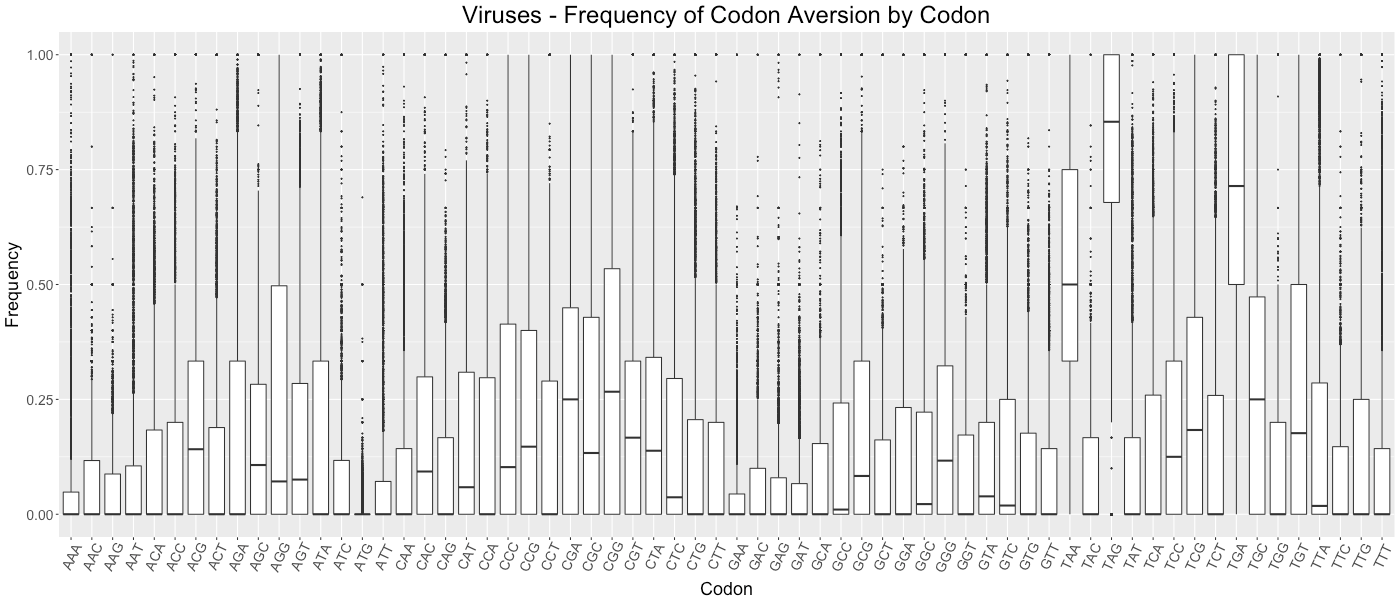

Supplement: Figure S21 — The frequency of codon exclusion for the taxonomic group. The box plot represents the frequency of species in the taxonomic group that exclude a certain codon in their genes (e.g., if a codon is not used in 50% of a species’ genes, then that species would be plotted at 0.50). [file peerj-07-6984-s023.png]

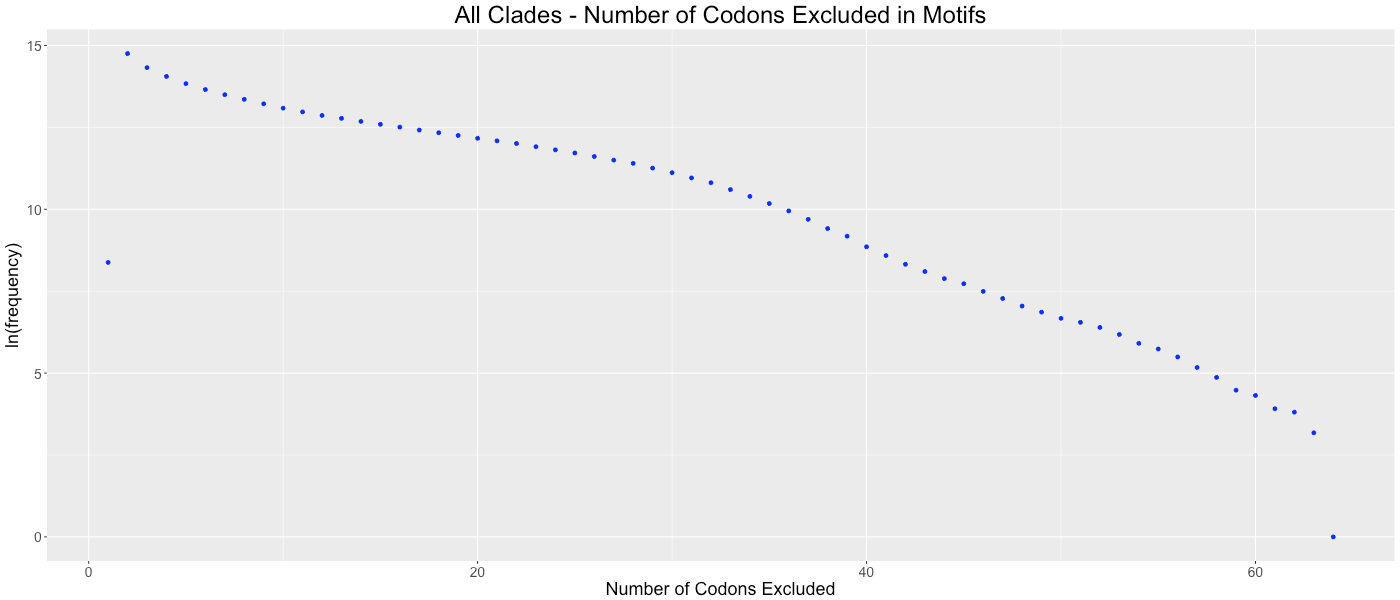

Supplement: Figure S22 — Shows the frequency of how many codons (0-64) are not used in each gene. [file peerj-07-6984-s024.png]

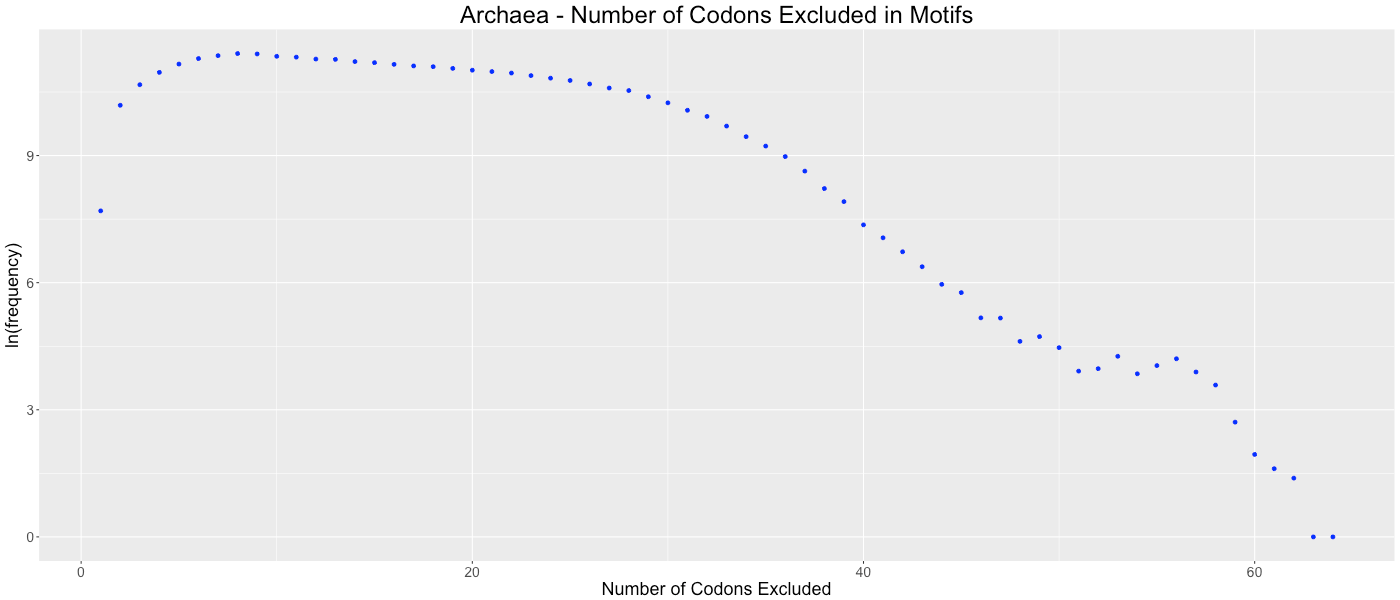

Supplement: Figure S23 — Shows the frequency of how many codons (0-64) are not used in each gene. [file peerj-07-6984-s025.png]

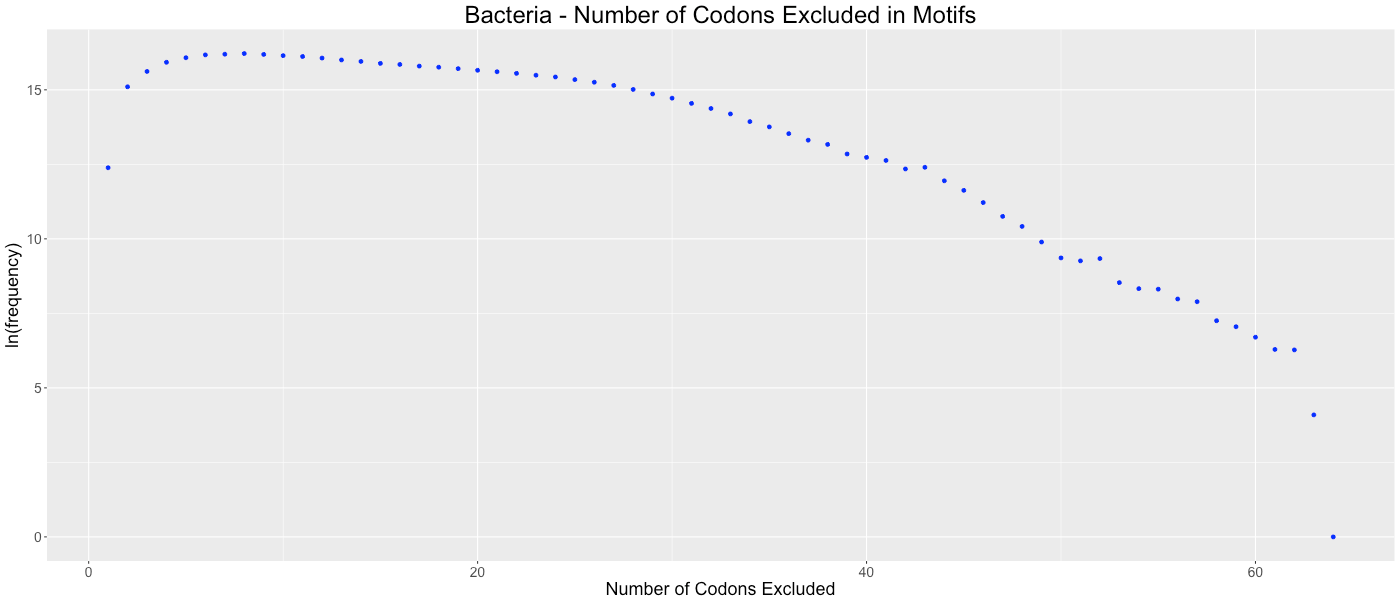

Supplement: Figure S24 — Shows the frequency of how many codons (0-64) are not used in each gene. [file peerj-07-6984-s026.png]

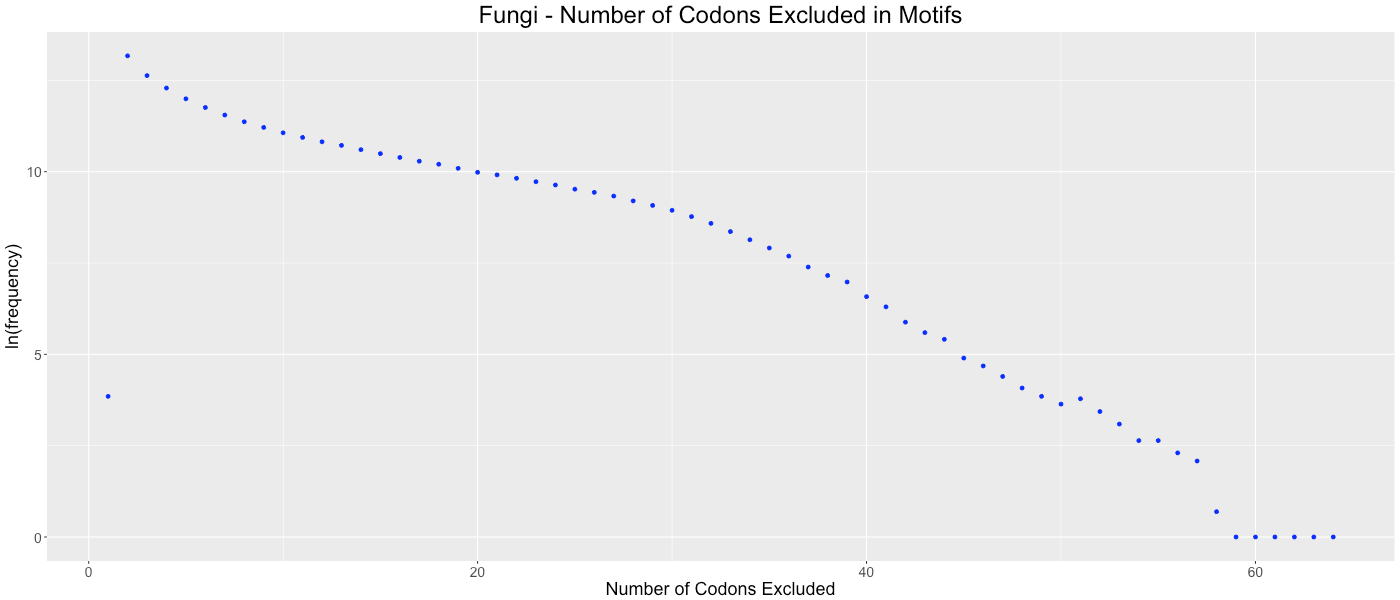

Supplement: Figure S25 — Shows the frequency of how many codons (0-64) are not used in each gene. [file peerj-07-6984-s027.png]

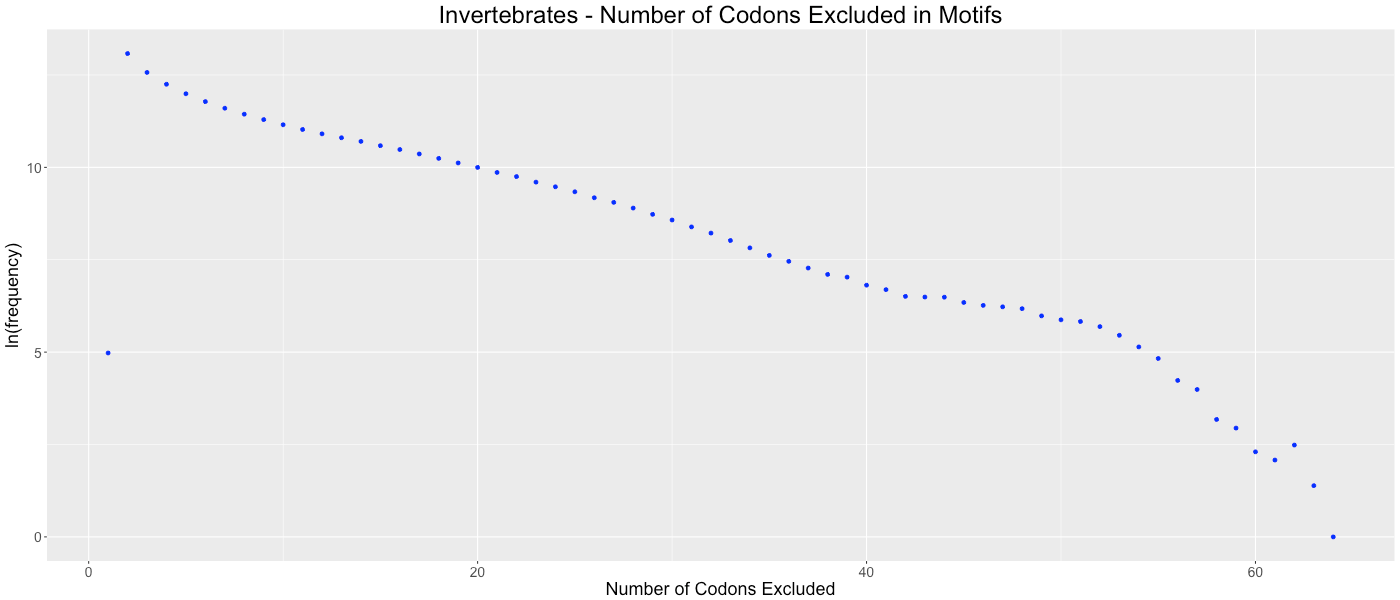

Supplement: Figure S26 — Shows the frequency of how many codons (0-64) are not used in each gene. [file peerj-07-6984-s028.png]

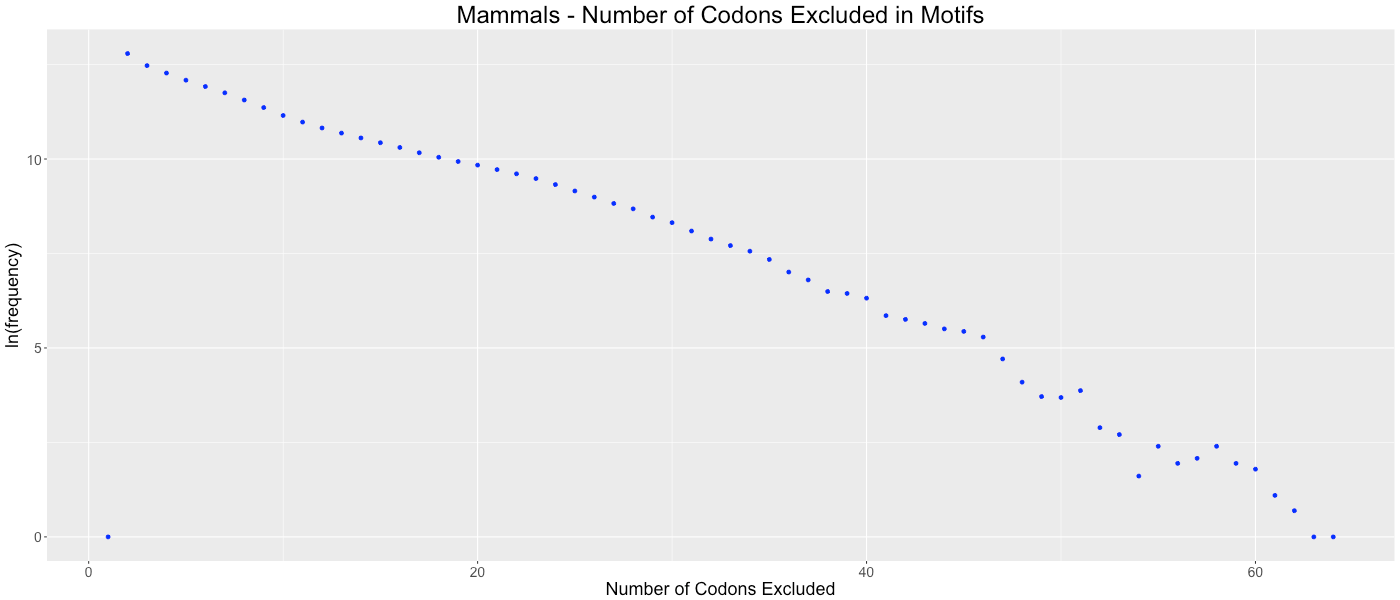

Supplement: Figure S27 — Shows the frequency of how many codons (0-64) are not used in each gene. [file peerj-07-6984-s029.png]

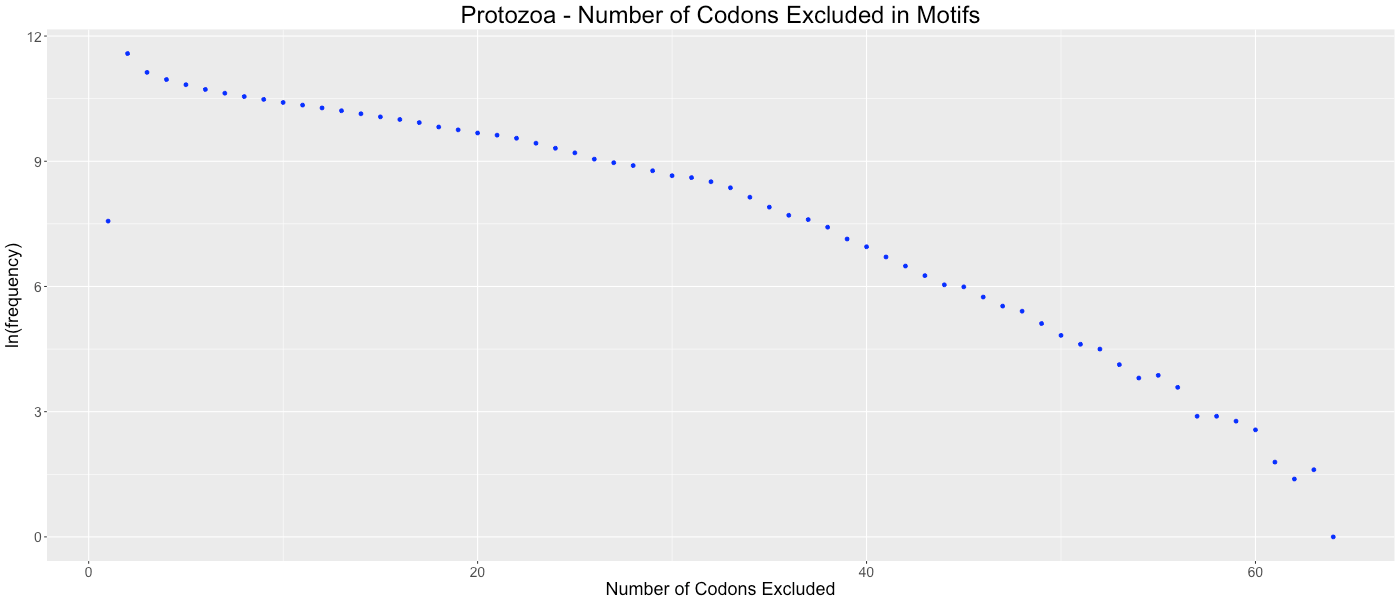

Supplement: Figure S28 — Shows the frequency of how many codons (0-64) are not used in each gene. [file peerj-07-6984-s030.png]

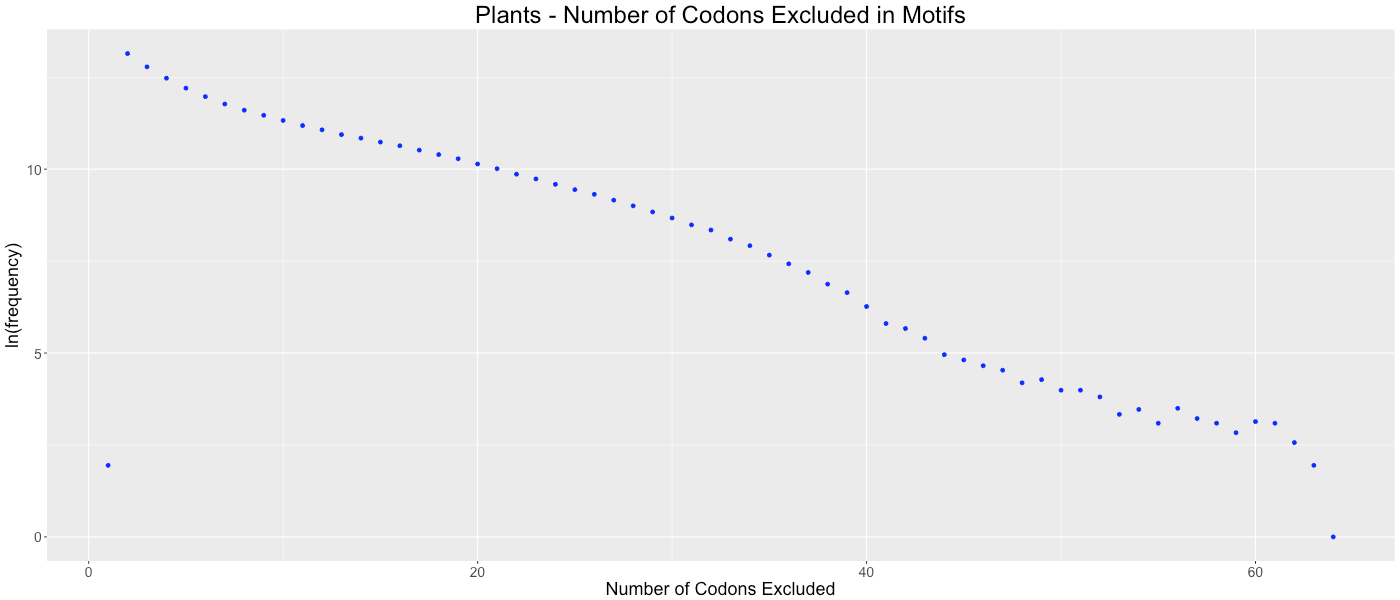

Supplement: Figure S29 — Shows the frequency of how many codons (0-64) are not used in each gene. [file peerj-07-6984-s031.png]

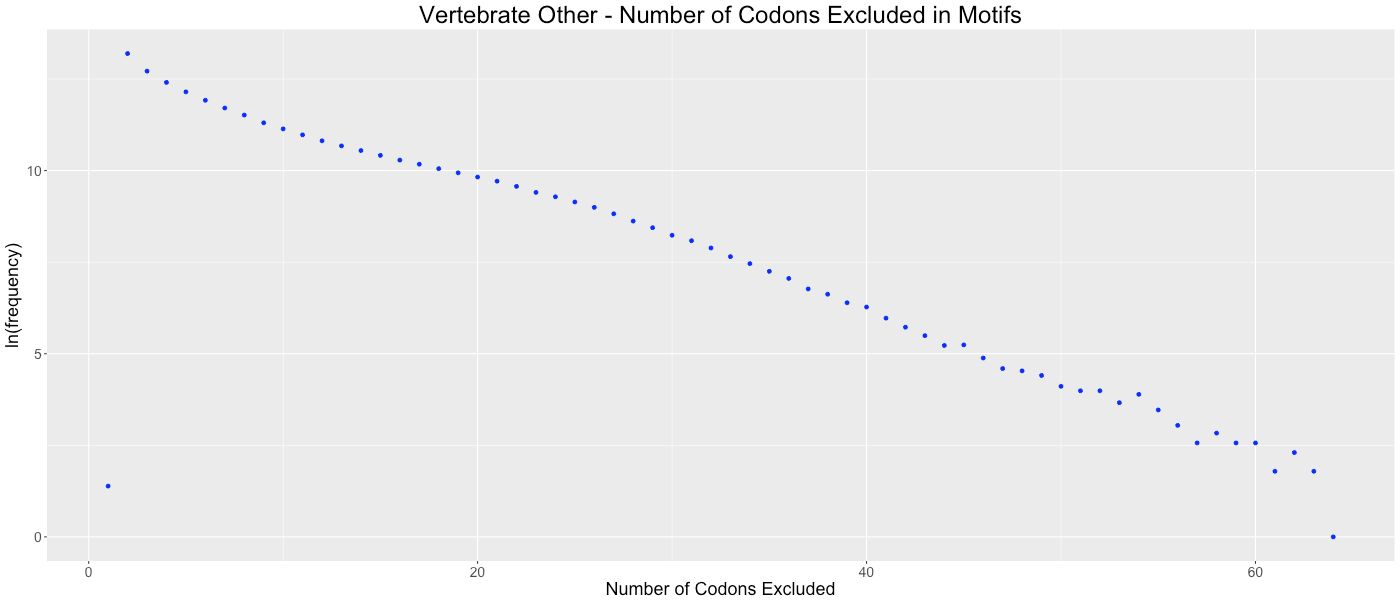

Supplement: Figure S30 — Shows the frequency of how many codons (0-64) are not used in each gene. [file peerj-07-6984-s032.png]

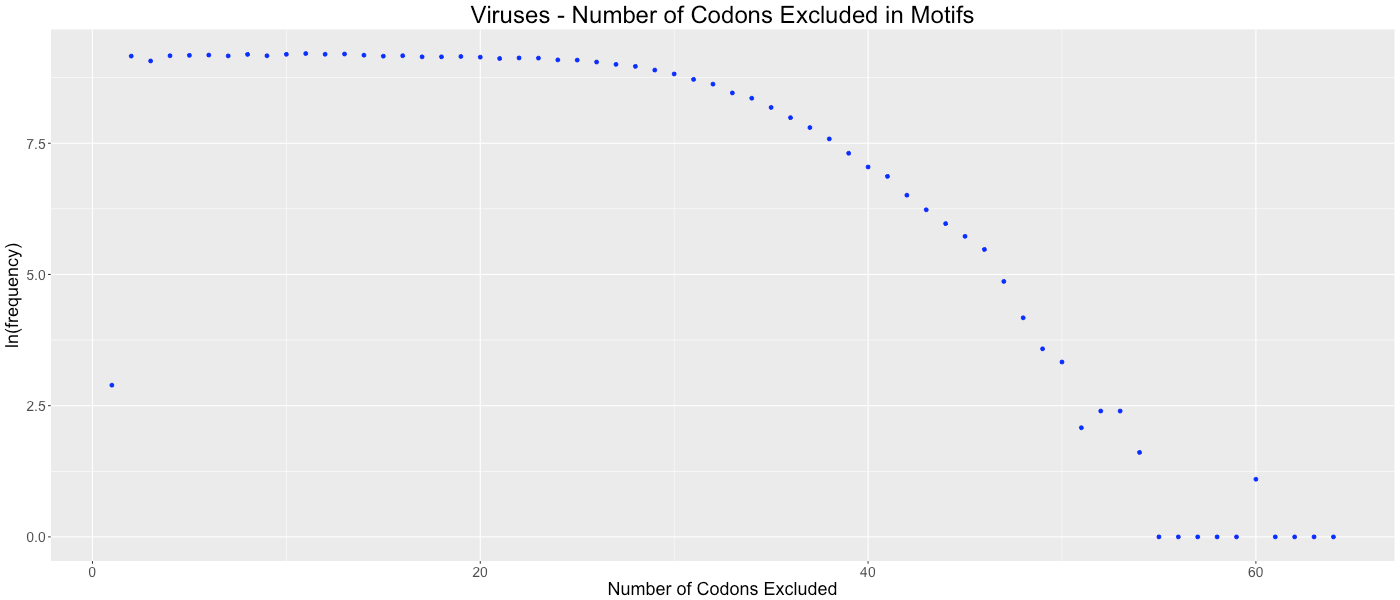

Supplement: Figure S31 — Shows the frequency of how many codons (0-64) are not used in each gene. [file peerj-07-6984-s033.png]

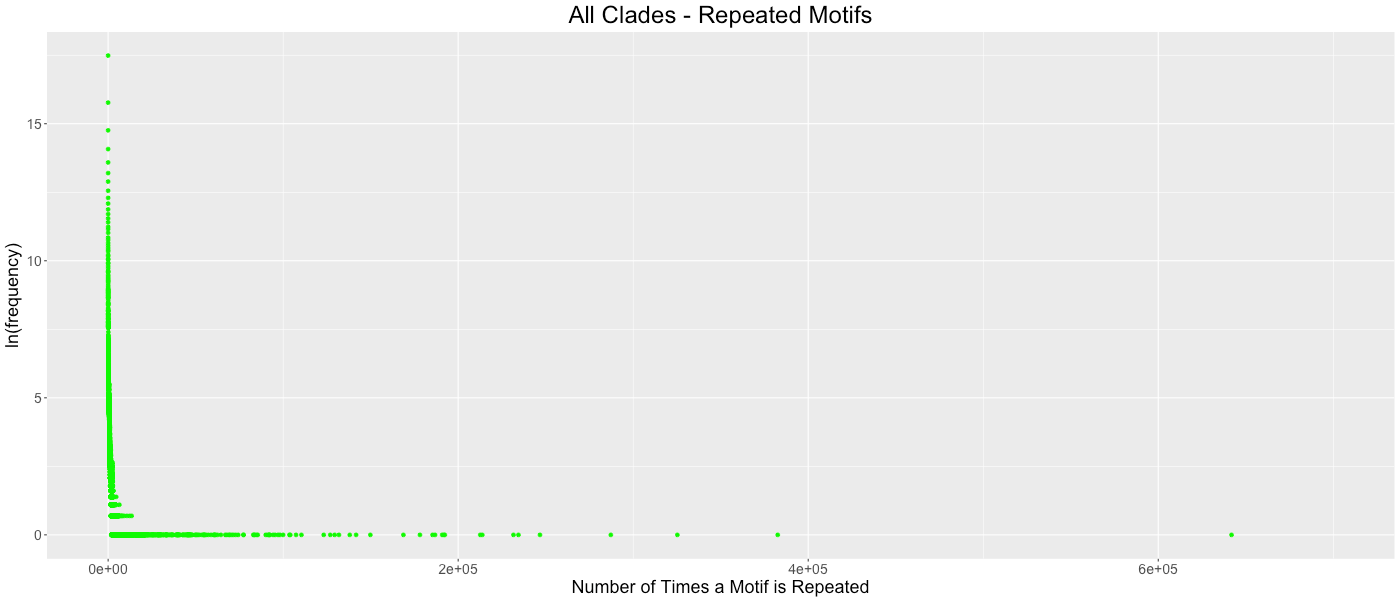

Supplement: Figure S32 — The frequency of which codon motifs are repeated is shown. The x- axis depicts how many time a motif was repeated in all the genes in a clade. The y-axis depicts how many motifs were repeated a given number of times (shown in the natural log). Some outliers were removed from each graph for clarity. These outliers represent the motifs in which only stop codons are excluded. All clades outliers excluded: (1309911,1), (2185083,1), (2433089,1). [file peerj-07-6984-s034.png]

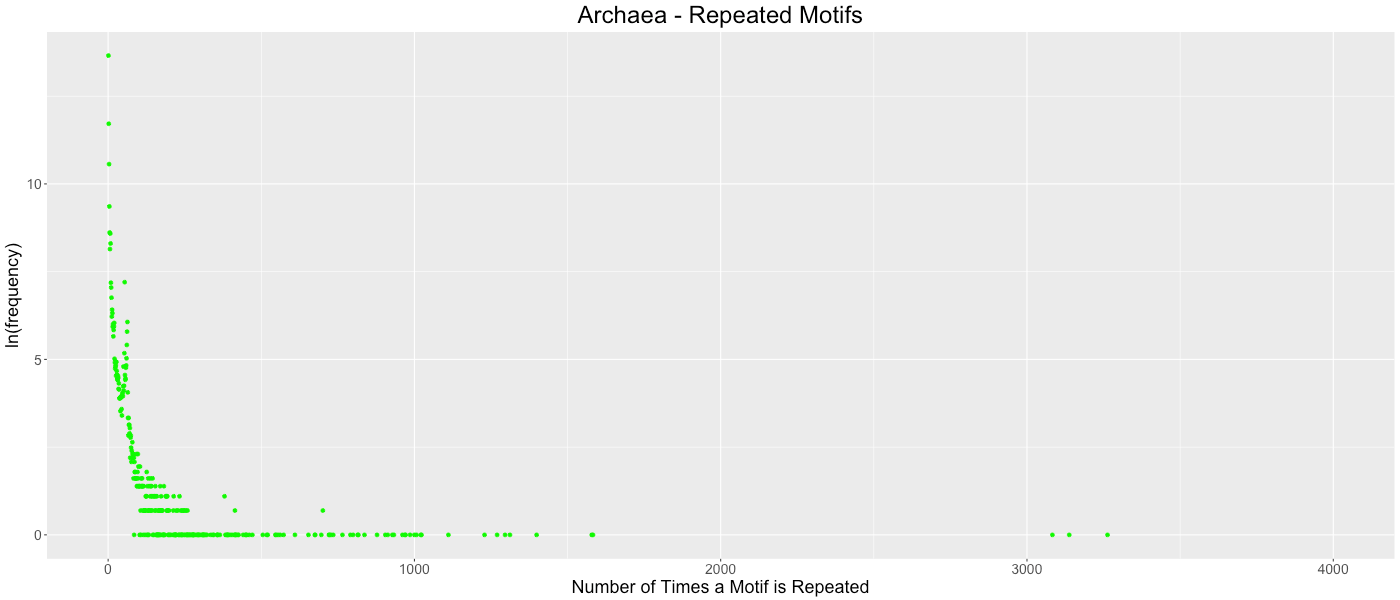

Supplement: Figure S33 — The frequency of which codon motifs are repeated is shown. The x- axis depicts how many time a motif was repeated in all the genes in a clade. The y-axis depicts how many motifs were repeated a given number of times (shown in the natural log). Some outliers were removed from each graph for clarity. These outliers represent the motifs in which only stop codons are excluded. Archaea outliers excluded: (10360,1), (10564,1). [file peerj-07-6984-s035.png]

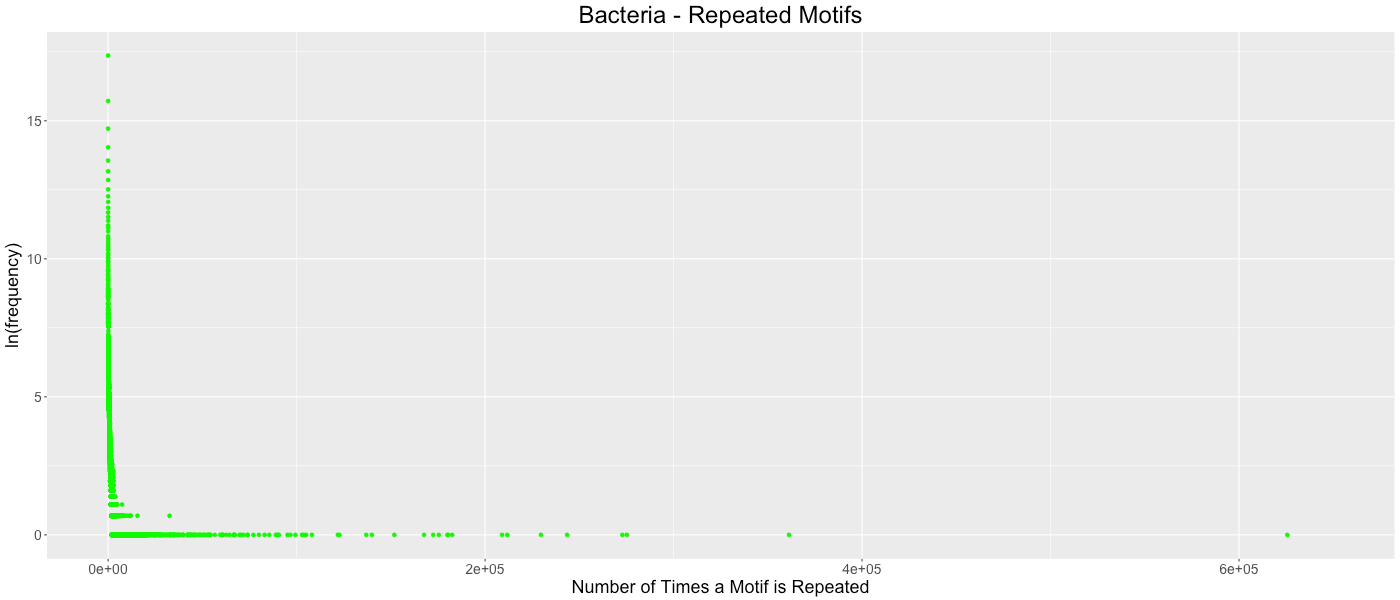

Supplement: Figure S34 — The frequency of which codon motifs are repeated is shown. The x- axis depicts how many time a motif was repeated in all the genes in a clade. The y-axis depicts how many motifs were repeated a given number of times (shown in the natural log). Some outliers were removed from each graph for clarity. These outliers represent the motifs in which only stop codons are excluded. Bacteria outliers excluded: (681998,1), (1085854,1), (1611727,1). [file peerj-07-6984-s036.png]

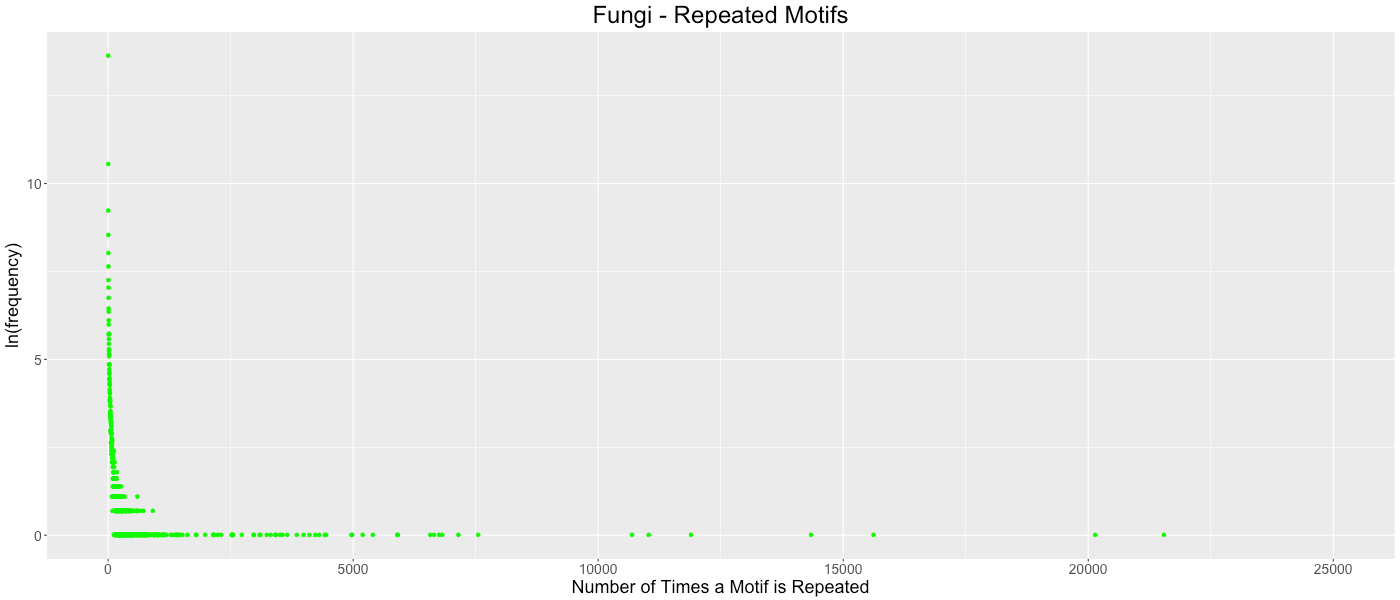

Supplement: Figure S37 — The frequency of which codon motifs are repeated is shown. The x- axis depicts how many time a motif was repeated in all the genes in a clade. The y-axis depicts how many motifs were repeated a given number of times (shown in the natural log). Some outliers were removed from each graph for clarity. These outliers represent the motifs in which only stop codons are excluded. Fungi outliers excluded: (140907,0), (157884,1), (226451,1). [file peerj-07-6984-s037.png]

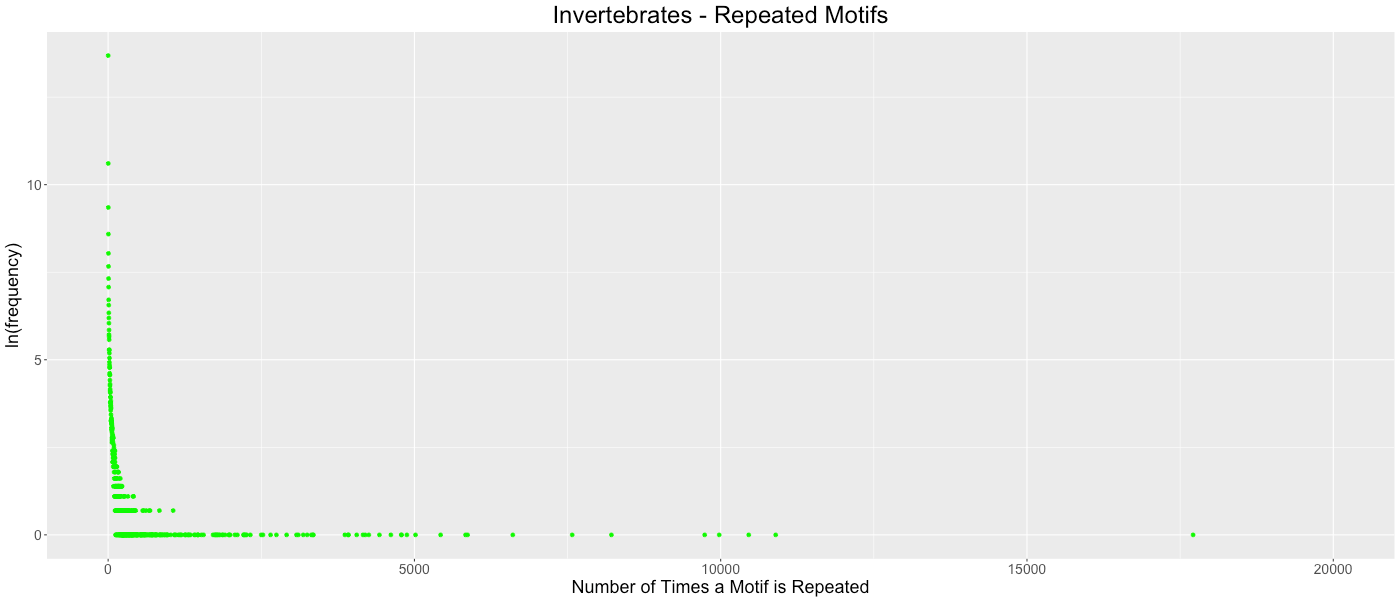

Supplement: Figure S36 — The frequency of which codon motifs are repeated is shown. The x- axis depicts how many time a motif was repeated in all the genes in a clade. The y-axis depicts how many motifs were repeated a given number of times (shown in the natural log). Some outliers were removed from each graph for clarity. These outliers represent the motifs in which only stop codons are excluded. Invertebrates outliers excluded: (110662,1), (201864,1), (166597,1). [file peerj-07-6984-s038.png]

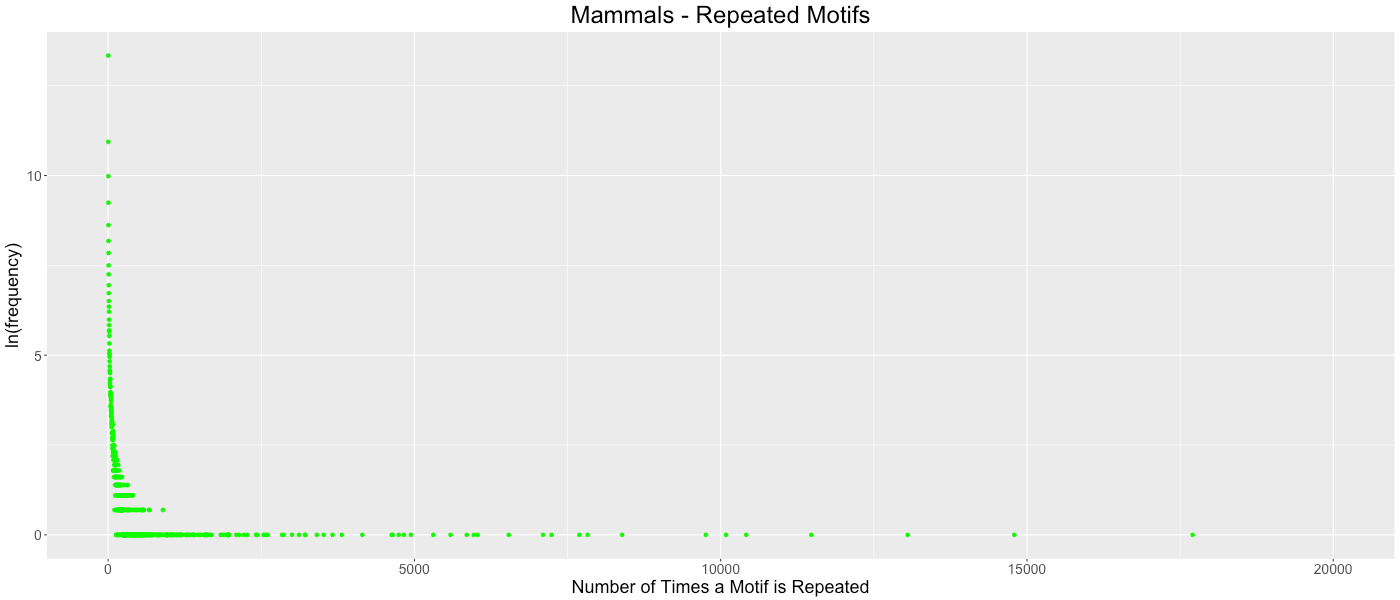

Supplement: Figure S37 — The frequency of which codon motifs are repeated is shown. The x- axis depicts how many time a motif was repeated in all the genes in a clade. The y-axis depicts how many motifs were repeated a given number of times (shown in the natural log). Some outliers were removed from each graph for clarity. These outliers represent the motifs in which only stop codons are excluded. Mammal outliers excluded: (81051,1), (105156,1), (17812,1). [file peerj-07-6984-s039.png]

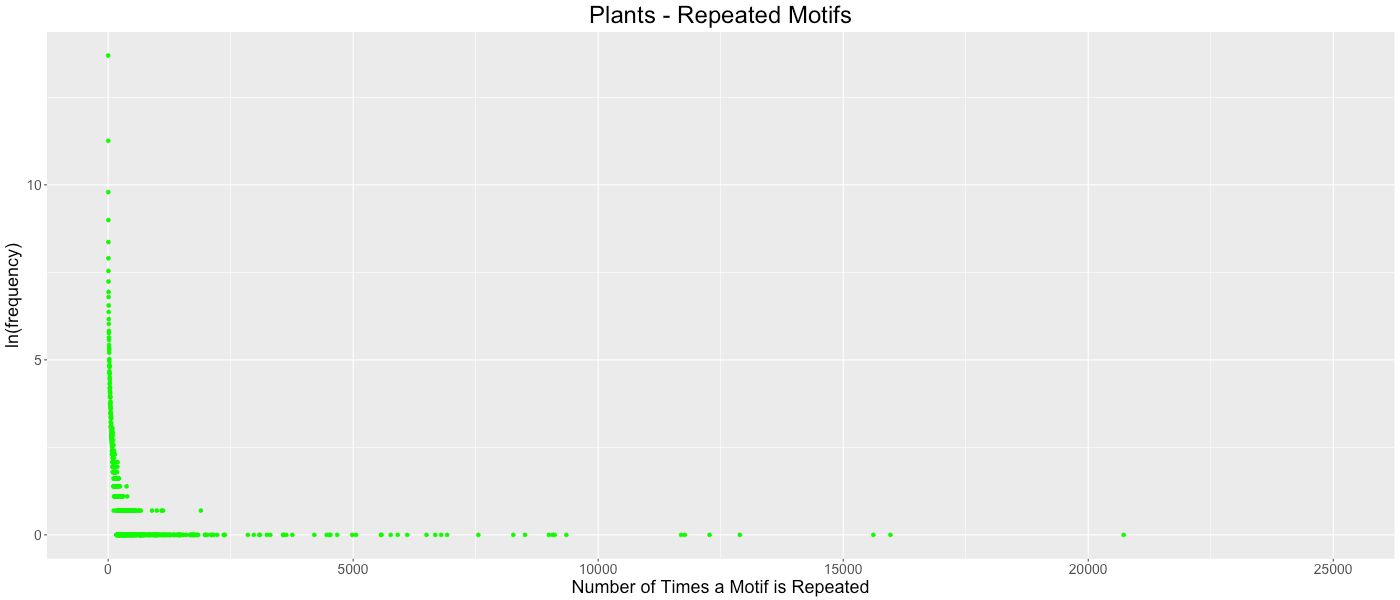

Supplement: Figure S38 — The frequency of which codon motifs are repeated is shown. The x- axis depicts how many time a motif was repeated in all the genes in a clade. The y-axis depicts how many motifs were repeated a given number of times (shown in the natural log). Some outliers were removed from each graph for clarity. These outliers represent the motifs in which only stop codons are excluded. Plants outliers excluded: (158430,1), (127795,1), (224688,1). [file peerj-07-6984-s040.png]

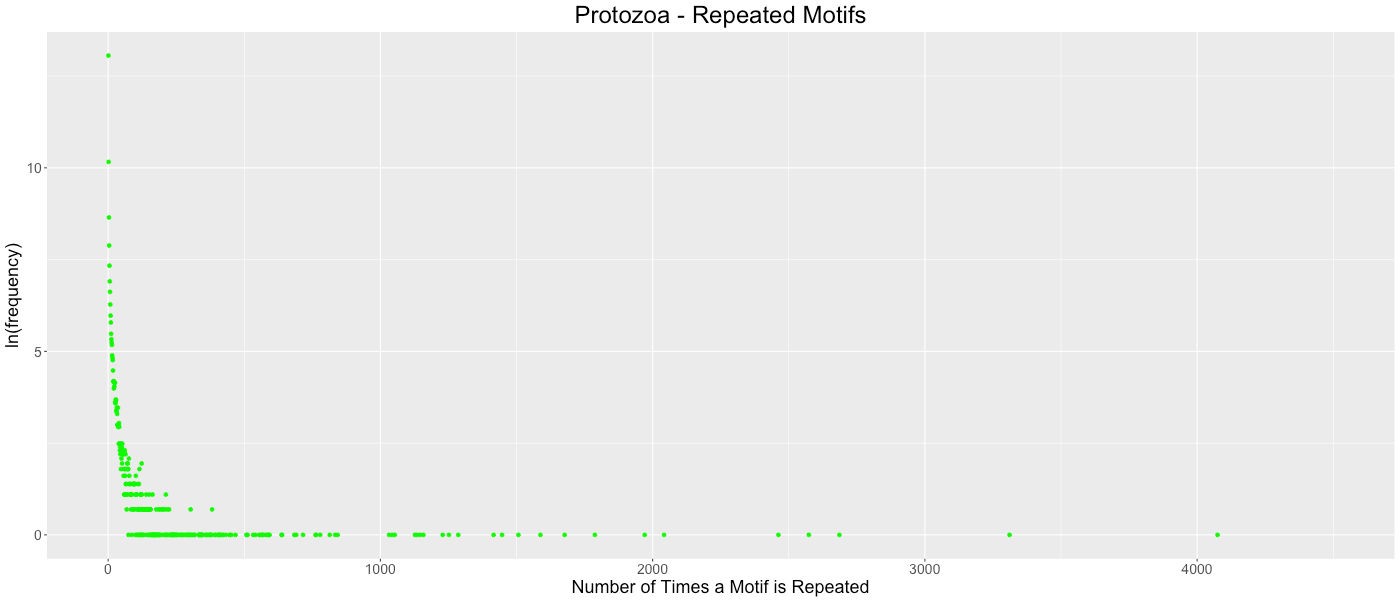

Supplement: Figure S39 — The frequency of which codon motifs are repeated is shown. The x- axis depicts how many time a motif was repeated in all the genes in a clade. The y-axis depicts how many motifs were repeated a given number of times (shown in the natural log). Some outliers were removed from each graph for clarity. These outliers represent the motifs in which only stop codons are excluded. Protozoa Outliers excluded: (32139,1), (41048,1), (30539,1). [file peerj-07-6984-s041.png]

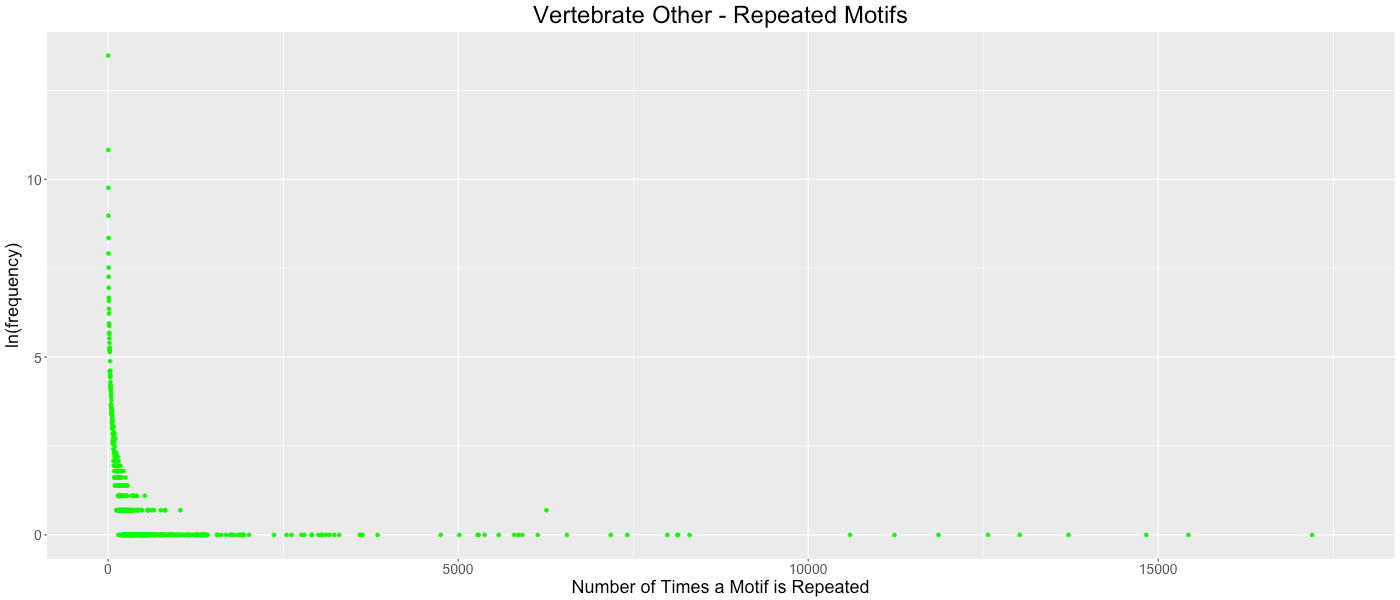

Supplement: Figure S40 — The frequency of which codon motifs are repeated is shown. The x- axis depicts how many time a motif was repeated in all the genes in a clade. The y-axis depicts how many motifs were repeated a given number of times (shown in the natural log). Some outliers were removed from each graph for clarity. These outliers represent the motifs in which only stop codons are excluded. Vertebrate other outliers excluded: (167892,1), (114746,1), (254804,1). [file peerj-07-6984-s042.png]

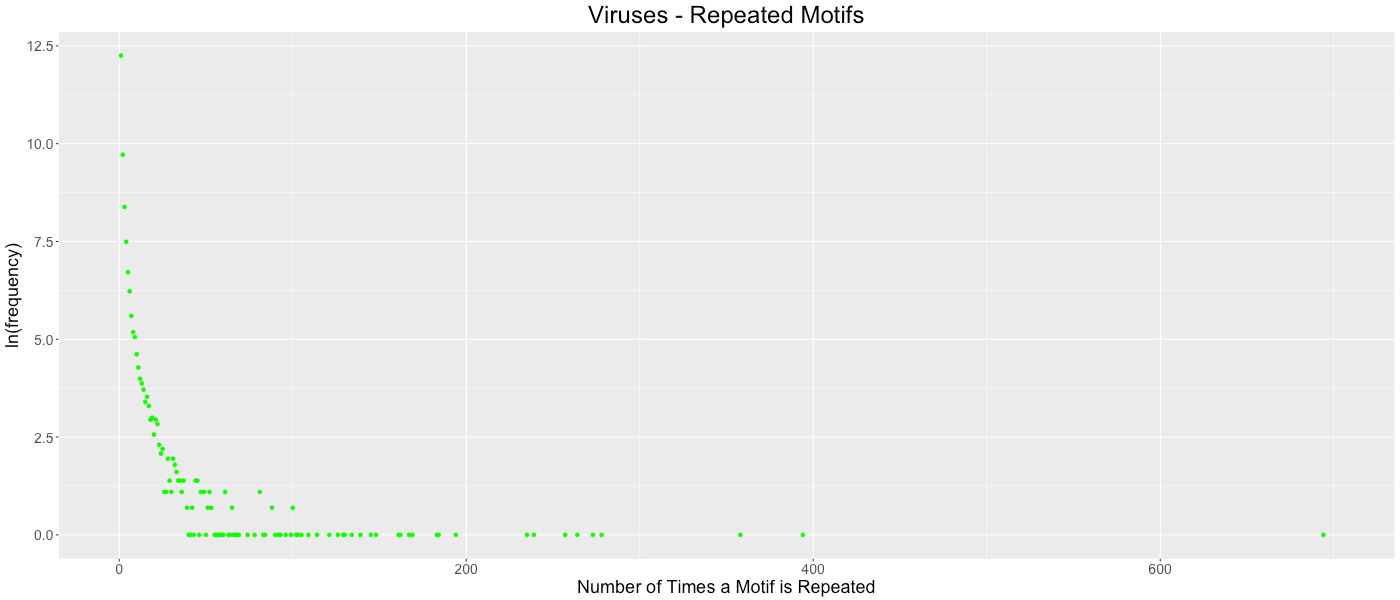

Supplement: Figure S41 — The frequency of which codon motifs are repeated is shown. The x- axis depicts how many time a motif was repeated in all the genes in a clade. The y-axis depicts how many motifs were repeated a given number of times (shown in the natural log). Some outliers were removed from each graph for clarity. These outliers represent the motifs in which only stop codons are excluded. Virus outliers excluded: (2669,1), (2167,1), (4664,1) [file peerj-07-6984-s043.png]
